# Compound C Prevents the Unfolded Protein Response during Glucose Deprivation through a Mechanism Independent of AMPK and BMP Signaling

**DOI:** 10.1371/journal.pone.0045845

**Published:** 2012-09-24

**Authors:** Sakae Saito, Aki Furuno, Junko Sakurai, Hae-Ryong Park, Kazuo Shin-ya, Akihiro Tomida

**Affiliations:** 1 Cancer Chemotherapy Center, Japanese Foundation for Cancer Research, Tokyo, Japan; 2 Department of Food Science and Biotechnology, Kyungnam University, Masan, Korea; 3 Biological Information Research Center, National Institute of Advanced Industrial Science and Technology, Tokyo, Japan; Texas A&M University, United States of America

## Abstract

Inhibiting the unfolded protein response (UPR) can be a therapeutic approach, especially for targeting the tumor microenvironment. Here, we show that compound C (also known as dorsomorphin), a small-molecule inhibitor of AMP-activated protein kinase (AMPK) and bone morphogenetic protein (BMP) signaling, inhibit the UPR-induced transcription program depending on the glucose deprivation conditions. We found that compound C prevented UPR marker glucose-regulated protein 78 (GRP78) accumulation and exerted enhanced cytotoxicity during glucose deprivation. Gene expression profiling, together with biochemical analysis, revealed that compound C had a unique mode of action to suppress the transcriptional activation of UPR-targeted genes, as compared with the classic UPR inhibitors versipelostatin and biguanides. Surprisingly, the UPR-inhibiting activity of compound C was not associated with either AMPK or BMP signaling inhibition. We further found that combination treatments of compound C and the classic UPR inhibitors resulted in synergistic cell death with UPR suppression during glucose deprivation. Our findings demonstrate that compound C could be a unique tool for developing a UPR-targeted antitumor therapy.

## Introduction

Glucose deprivation is a common feature of the solid tumor microenvironment and is caused by a combination of the poorly formed tumor vasculature, uncontrolled proliferation and abnormal energy metabolism of cancer cells. As does hypoxia, glucose deprivation leads to the abnormal accumulation of protein within the endoplasmic reticulum (ER), which triggers the activation of the unfolded protein response (UPR) in tumor cells [1], [2]. The UPR in cancer cells plays an important role in their survival under stress conditions and results in tumor malignancies and in antitumor drug resistance, whereas, in the case of intolerable levels of ER stress, the UPR can contribute to eliciting apoptosis [1], [2], [3]. Thus, the UPR is a potential target of antitumor therapy, and the repression or induction of the UPR by drugs may have therapeutic effects against tumors.

The UPR consists of three main signaling pathways initiated by ER membrane-localized stress sensors, PKR-like ER kinase (PERK), activating transcription factor 6 (ATF6) and inositol-requiring 1 (IRE1) [1], [3]. PERK induces the transcription factor activating transcription factor 4 (ATF4) through the phosphorylation of eukaryotic translation initiation factor 2 subunit alpha (eIF2α), which also transiently leads to attenuation of global translation [4], [5], [6]. ATF6 becomes an active transcription factor by proteolytic cleavage [7], [8], whereas IRE1 mediates the unconventional splicing of X-box binding protein 1 (XBP1) mRNA, thereby converting it to a potent UPR transcriptional activator [9], [10], [11], [12]. These transcription factors lead to coordinated induction of divergent UPR target genes, such as the ER-resident molecular chaperones glucose-regulated protein 78 and 94 (GRP78 and GRP94), for cell survival [13].

We previously reported that a novel macrocyclic compound versipelostatin and antidiabetic biguanides (phenformin, metformin and buformin) prevented the UPR and exerted highly selective cytotoxicity in glucose-deprived cancer cells [14], [15]. These drugs inhibit production of the UPR transcription activators ATF6, ATF4 and XBP1 and broadly suppress the transcription program of the glucose deprivation–induced UPR. This UPR inhibition is partly mediated by the aberrant hyperactivation of eukaryotic initiation factor 4E-binding protein 1 (4E-BP1) [16]. We also found that mitochondria dysfunction leads to failure of UPR activation depending on the glucose deprivation conditions [17], suggesting that the glucose deprivation–induced UPR is governed by unique regulatory mechanisms, which is not affected by tunicamycin or other chemical stressors. Of note is that versipelostatin, metformin and phenformin exert *in vivo* antitumor activity [14], [18], [19], demonstrating the potential of UPR inhibition as an attractive anticancer approach.

In the course of screening for UPR inhibitors, we found that compound C (6-[4-(2-Piperidin-1-yl-ethoxy)-phenyl)]-3-pyridin-4-yl-pyrazolo[1,5-a]-pyrimidine), also known as dorsomorphin, could inhibit activation of a GRP78 promoter reporter in cancer cells during glucose deprivation. Compound C is a kinase inhibitor developed in the search for small-molecule inhibitor of AMP-activated protein kinase (AMPK) [20]. Compound C reversibly and directly inhibits AMPK activation and is competitive with ATP. Recently, compound C has also been found to inhibit the bone morphogenetic protein (BMP) type I receptors, the activin-like kinase receptor 2, 3, and 6 (ALK2, ALK3 and ALK6), independently of AMPK inhibition [21].

Here we demonstrate that compound C inhibits the UPR in glucose-deprived tumor cells independently of AMPK and BMP signaling. The modes of action of compound C are different from the previously identified, classic UPR inhibitors versipelostatin and the biguanides, as shown by gene expression profiling and biochemical analysis. We also show that combinations of compound C and the classic UPR inhibitors synergistically prevent the UPR and kill cancer cells during glucose deprivation.

## Results

### Compound C Inhibits GRP78 Induction During Glucose Deprivation

**Figure 1:**
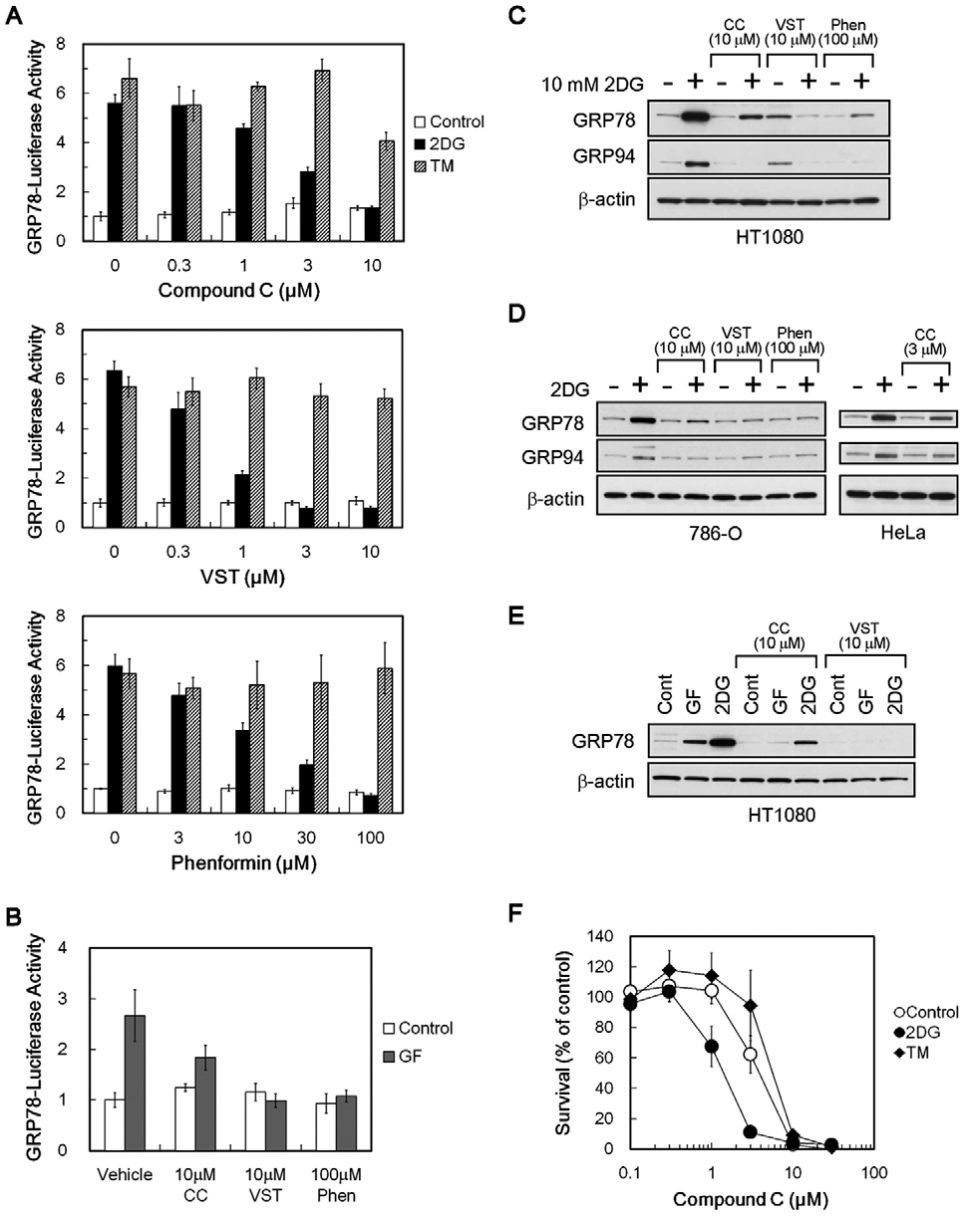
Suppressive effects of compound C on GRP78 and GRP94 protein accumulation. (A, B) Reporter assay. HT1080 cells were transfected with pGRP78pro160-Luc and exposed to stress (2DG, 10 mM 2-deoxy-D-glucose; TM, 5 µg/mL of tunicamycin (A) or GF, glucose-free (B)) for 18 h with compound C, versipelostatin or phenformin. Results shown are the means ± SD of quadruplicate determinations. (C–E) Immunoblot analysis. In C, HT1080 cells were treated for 18 h with compound C, versipelostatin or phenformin in the presence (+) or absence (−) of 10 mM 2DG. In D, 786-O cells (*left*) were treated for 18 h with compound C, versipelostatin or phenformin in the presence (+) or absence (−) of 10 mM 2DG. HeLa cells (*right*) were treated for 15 h with compound C in the presence (+) or absence (−) of 5 mM 2DG. Because HeLa cells exhibited hypersensitivity to cytocidal action of compound C during glucose deprivation, it was necessary to reduce the concentrations of 2DG and compound C, as compared with HT1080 and 786-O cell analysis. In E, HT1080 cells were treated for 18 h with compound C or versipelostatin in normal medium (Cont), normal medium with 10 mM 2DG (2DG) or glucose-free medium (GF). CC, compound C; VST, versipelostatin; Phen, phenformin. β-actin was used as a loading control. (F) MTT assay of HeLa cells treated with compound C under normal or ER stress conditions. 2DG, 10 mM 2-deoxyglucose; TM, 1 µg/mL of tunicamycin. Results shown are the means ± SD.

We first examined the effects of compound C on UPR marker GRP78 promoter activity in human fibrosarcoma HT1080 cells that were transiently transfected with the reporter gene plasmid pGRP78pro160-Luc [14]. We used two different types of chemical UPR inducers, the hypoglycemia-mimicking agent 2-deoxy-D-glucose (2DG) and the *N*-glycosylation inhibitor tunicamycin. As shown in Figure 1A, treating the transfected cells with 2DG and tunicamycin increased GRP78 promoter activity by approximately 6-fold. Similarly to versipelostatin and phenformin, compound C suppressed 2DG-induced GRP78 promoter activity in a dose-dependent manner but had little effect on tunicamycin-induced GRP78 promoter activity. Compound C also suppressed GRP78 promoter activity induced by glucose withdrawal (Figure 1B), indicating that 2DG addition and glucose withdrawal were equivalent for compound C to exert UPR-inhibitory activity, although the intensity of GRP78 induction was somewhat different between each of the stress condition (*see also* Figure 1E).

We next examined the effects of compound C on endogenous ER-resident molecular chaperone proteins GRP78 and GRP94 by immunoblot analysis. As shown in Figure 1C, compound C suppressed accumulation of endogenous GRP78 and GRP94 proteins in HT1080 under 2DG stress conditions. In agreement with our previous study [14], versipelostatin slightly induced GRP78 and GRP94 protein under normal growth conditions, but compound C did not show such an activity in HT1080 cells (Figure 1C), suggesting that the mechanisms of action of versipelostatin, but not compound C, may be changeable under normal growth conditions and under glucose deprivation conditions. Inhibition of 2DG-induced accumulation of GRP78 and GRP94 proteins by compound C was also observed in renal cell carcinoma 786-O cells and cervical carcinoma HeLa cells (Figure 1D). Compound C also suppressed GRP78 protein accumulation in HT1080 during glucose withdrawal (Figure 1E). Consistent with the previous observation of versipelostatin and phenformin [14], cytotoxicity of compound C was enhanced under 2DG stress conditions but not under tunicamycin stress (Figure 1F).

### Compound C uses a Unique Mode of Action to Inhibit the UPR

**Figure 2:**
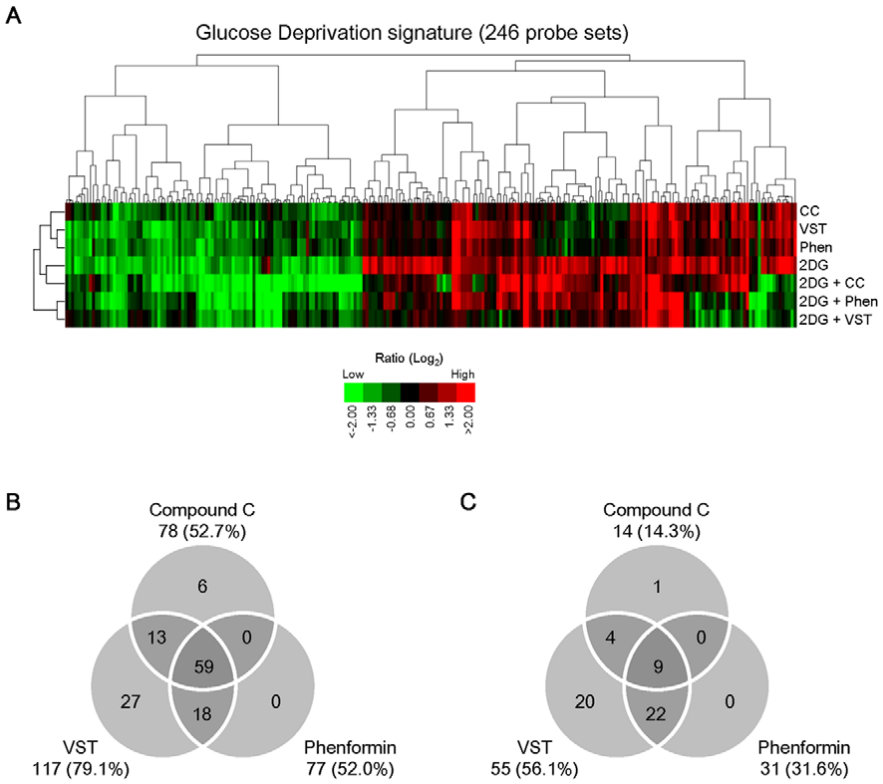
Inhibition of glucose deprivation-induced gene expression by compound C. (A) Glucose deprivation signature, including 246 probe sets (*X* axis) sorted by cluster analysis, displayed with 7 samples (*Y* axis). HT1080 cells were cultured with 10 µM compound C, 10 µM versipelostatin and 100 µM phenformin for 18 h in the presence or absence of 10 mM 2DG. The log ratio for each gene was calculated by setting the expression level in appropriate control sample (non­–drug-treated cells) as 0 (Log_2_ 1). Details of the experimental conditions are provided as Table S1. 2DG, 2-deoxy-D-glucose; CC, compound C; VST, versipelostatin; Phen, phenformin. (B, C) Effects of UPR inhibitors on Glucose Deprivation signature genes. The inhibition efficiency of compound C, versipelostatin and phenformin under 2DG stress conditions was expressed using the relative fold change ratio for each of 246 probe sets, as determined by setting each 2DG-induced fold change in gene expression levels at 100%. Venn diagram shows the number of unique and shared probe sets, which indicated the inhibition efficiency ≧50% in the up-regulated 148 probe sets (B) or down-regulated 98 probe sets (C) of the Glucose Deprivation signature.

To further characterize the UPR-inhibitory activity of compound C, we carried out gene expression profiling of HT1080 cells treated with compound C, versipelostatin, or phenformin under normal or 2DG stress conditions. The gene expression analysis showed that compound C broadly inhibited the 2DG-inducible genes in the Glucose Deprivation Signature (Figure 2 and Table S2, S3), which we identified previously as a UPR-related gene set under glucose deprivation conditions [15]. The genes for which expression was inhibited by compound C considerably overlapped with the genes for which expression was suppressed by the classic inhibitors versipelostatin and phenformin (Figure 2A–C). Nevertheless, the inhibitory pattern of the Glucose Deprivation Signature by compound C appeared different from the versipelostatin and phenformin patterns. The overall expression profile of compound C was also different from those of versipelostatin and phenformin under normal or 2DG stress conditions (Figure S1).

**Figure 3:**
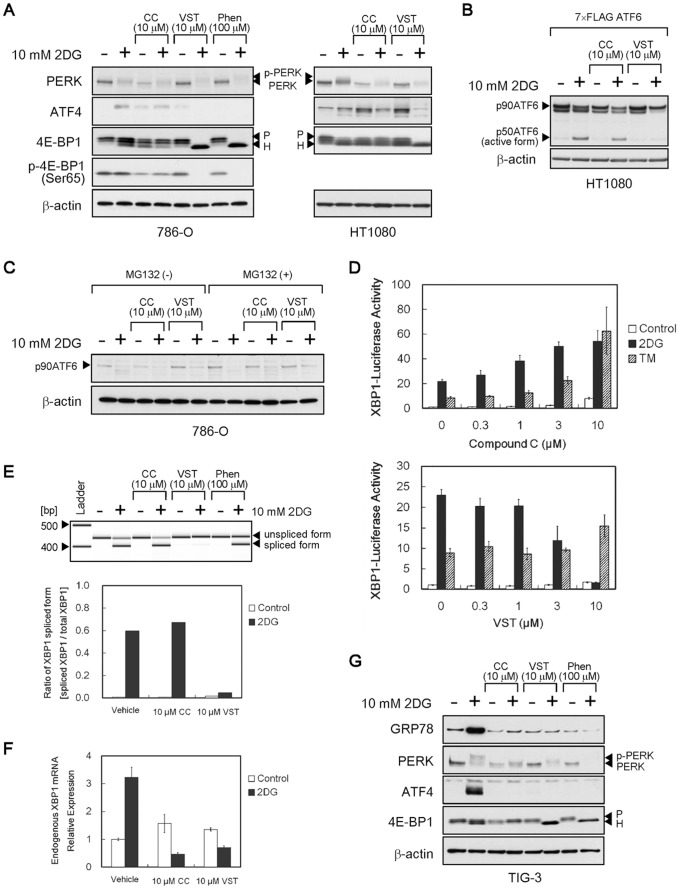
Effects of compound C on the UPR signaling pathway. (**A**–**C**) **Immunoblot analysis of UPR-related proteins.** In A, HT1080 (*right*) and 786-O cells (*left*) were treated for 18 h with compound C, versipelostatin or phenformin in the presence (+) or absence (−) of 10 mM 2DG. In B, HT1080 cells were transfected with 100 ng of 7× FLAG-tagged ATF6 plasmid (expressed FLAG-tagged full-length p90ATF6) and treated for 6 h with compound C or versipelostatin in the presence (+) or absence (−) of 10 mM 2DG. For better detection of the p50ATF6/active form, MG132 was included during exposure of cells to 2DG. In C, 786-O cells were treated for 6 h with compound C and versipelostatin in the presence (+) or absence (−) of 10 mM 2DG and MG132. β-actin was used as a loading control. (D) XBP1 reporter assay. XBP1-Luc/HT1080 cells were transfected with phRL-CMV and exposed to stress (10 mM 2DG or 5 µg/mL of tunicamycin) for 18 h with compound C and versipelostatin. Results shown are the means ± SD of quadruplicate determinations. (E, F) Quantitative PCR (E) and real-time PCR (F) analysis of XBP1 transcript. HT1080 cells were treated with compound C and versipelostatin for 18 h under normal or 2DG stress conditions. In *E*, PCR products were analyzed by Agilent 2100 Bioanalyzer (gel-like image, *upper*). Relative ratios of XBP1 mRNA splicing valiant were calculated by setting each total expression amount of XBP1 mRNA in cells as 1 (graph, *lower*). Two independent experiments were performed to confirm the reproducibility. In F, relative expression levels of endogenous XBP1 mRNA were calculated by setting each normal expression level from non–drug-treated cells as 1. Results shown are the means ± SD. (G) Immunoblot analysis of UPR-related proteins. TIG-3 cells were treated for 18 h with compound C, versipelostatin or phenformin in the presence (+) or absence (−) of 10 mM 2DG. β-actin was used as a loading control. 2DG, 2-deoxy-D-glucose; TM, tunicamycin; CC, compound C; VST, versipelostatin; Phen, phenformin.

We next compared the effects of compound C with those of the classic inhibitors on the UPR signaling pathways that originated from PERK, ATF6 and IRE1 (Figure 3). Similar to versipelostatin and phenformin, compound C had no effect on 2DG-induced PERK activation (as shown by phosphorylation-mediated band shifts) in HT1080 and 786-O cells although it tended to decrease PERK expression levels under normal conditions (Figure 3A). As previously reported [15], [16], versipelostatin, as well as phenformin, suppressed 2DG-induced expression of the PERK-downstream transcription factor ATF4, which was accompanied by hyperactivation of 4E-BP1 (as shown by hypophosphorylation-mediated band shifts) (Figure 3A). However, compound C did not produce these events, which were associated with UPR inhibition by the classic inhibitors (Figure 3A). As to the signaling pathway of ATF6, we examined the proteolytic cleavage of overexpressed and endogenous ATF6 under 2DG stress conditions. In HT1080 cells, versipelostatin inhibited 2DG-induced ATF6 activation, but compound C did not (Figure 3B). In 786-O cells, whereas endogenous active/cleaved p50ATF6 was not detected, it was observed that p90ATF6 decreased as a result of cleavage activation under 2DG stress conditions (Figure 3C). Compound C did not inhibited 2DG-induced p90ATF6 protein reduction in 786-O cells. To examine the signaling pathway of IRE1, we used XBP1-Luc/HT1080 cells which stably expressed a reporter gene for monitoring the IRE1-splicing activity. Versipelostatin suppressed 2DG-induced, but not tunicamycin-induced, reporter activity, whereas compound C enhanced the reporter activity induced by both stressors (Figure 3D). Consistently, RT-PCR analysis revealed that compound C, but not versipelostatin, sustained the ratio of the IRE1-mediated, spliced form of XBP1 mRNA at high levels under 2DG stress conditions (Figure 3E), in spite of a decrease in the endogenous total XBP1 mRNA levels (Figure 3F). Collectively, these results indicate that compound C inhibited the UPR transcription program during glucose deprivation, possibly through a mechanism different from those of the classical UPR inhibitors versipelostatin and phenformin.

Next, we used human lung fibroblast TIG-3 cells to assess the effect of compound C on the UPR signaling pathways in non-tumor cell types. As shown in Figure 3G, treating TIG-3 cells with 2DG induced GRP78 protein accumulation and activation of the PERK and ATF4 signaling pathway. Similarly to versipelostatin and phenformin, compound C suppressed 2DG-induced accumulation of GRP78 and ATF4 proteins in TIG-3 cells under 2DG stress conditions. As observed in tumor cells, compound C also tended to decrease PERK expression levels under normal conditions and did not induce hyperactivation of 4E-BP1 under 2DG stress conditions. These results suggest that compound C as well as versipelostatin and phenformin can prevent the UPR activation during glucose deprivation in certain normal cells, in addition to tumor cells.

### AMPK is Dispensable for UPR Activation

**Figure 4:**
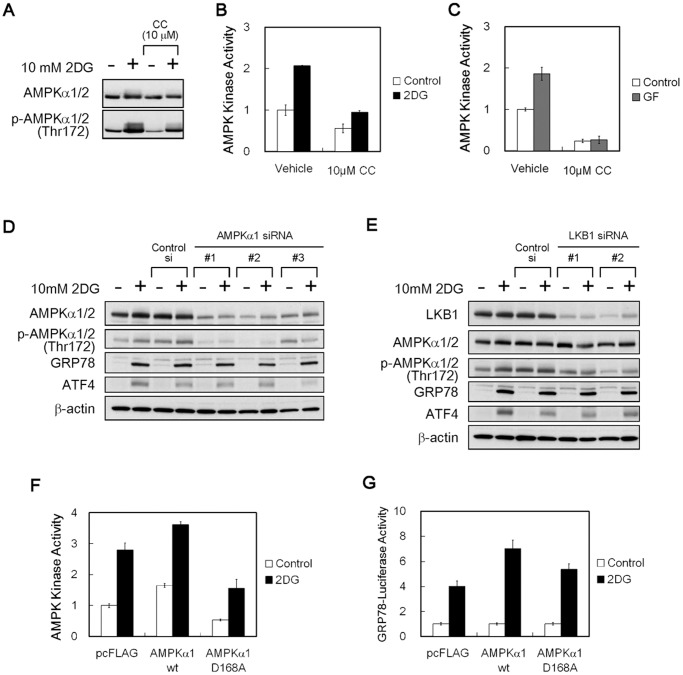
Effects of the AMPK kinase activity inhibition on GRP78 accumulation. (A) Immunoblot analysis. HT1080 cells were treated with compound C for 4 h in the presence (+) or absence (−) of 10 mM 2DG. β-actin was used as a loading control. (B, C) AMPK kinase assay. HT1080 cells were treated with compound C for 2 h under 2DG stress (B) or glucose withdrawal (C) conditions. CC, compound C; GF, glucose-free. Results shown are the means ± SD of triplicate determinations. (D, E) Immunoblot analysis. HT1080 cells were transfected with AMPKα1 (D) or LKB1 siRNA (E) and cultured for 18 h in the presence (+) or absence (−) of 10 mM 2DG. β-actin was used as a loading control. (F) AMPK kinase assay. HT1080 cells were transfected with plasmid subcloned wild-type AMPKα1 or dominant negative D168A, and cultured for 2 h under normal or 2DG stress conditions. A pcFLAG vector was used as a control. Results shown are the means ± SD of duplicate determinations. (G) Reporter assay. HT1080 cells were co-transfected with pGRP78pro160-Luc and plasmid-expressed wild-type AMPKα1 or dominant negative D168A and were cultured for 18 h under normal or 2DG stress conditions. A pcFLAG vector was used as a control. Relative ratios of 2DG-induced promoter activities were calculated by setting normal activation level in each cell as 1. Results shown are the means ± SD of quadruplicate determinations. 2DG, 10 mM 2-deoxy-D-glucose.

Compound C is known as an inhibitor of AMPK [20], and we examined whether compound C indeed affected AMPK activity during glucose deprivation. Immunoblot analysis revealed that phosphorylation levels of the catalytic α subunit of AMPK were increased by exposure of HT1080 cells to 2DG, whereas both basal and 2DG-induced phosphorylation levels were clearly reduced when compound C was added (Figure 4A, and Figure S2 for longer exposure periods). Measurements of cellular kinase activity using an ELISA-based assay system confirmed that compound C did reduce the endogenous AMPK activity regardless of cell culture conditions (Figure 4B, C).

To examine the possibility that AMPK inhibition might be involved in UPR inhibition, we used three approaches to modifying AMPK activity in HT1080 cells: AMPKα1 knockdown, LKB1, a major upstream kinase of AMPK [22], [23] knockdown, and overexpression of a dominant negative AMPKα1. AMPKα1 knockdown effectively reduced AMPKα protein but did not suppress GRP78 induction under 2DG stress conditions (Figure 4D). LKB1 knockdown effectively reduced AMPKα phosphorylation but did not prevent GRP78 induction (Figure 4E). Furthermore, we found that overexpression of the kinase-inactive form AMPKα1-D168A successfully reduced AMPK kinase activity in the cells by approximately 50% in the presence or absence of 2DG (Figure 4F) but did not inhibit GRP78 promoter activity (Figure 4G). These results indicate that AMPK was not required for UPR activation during glucose deprivation, although compound C could inhibit AMPK at the same concentrations necessary to inhibit the UPR.

### Inhibition Activities Against BMP and UPR are Different

**Figure 5:**
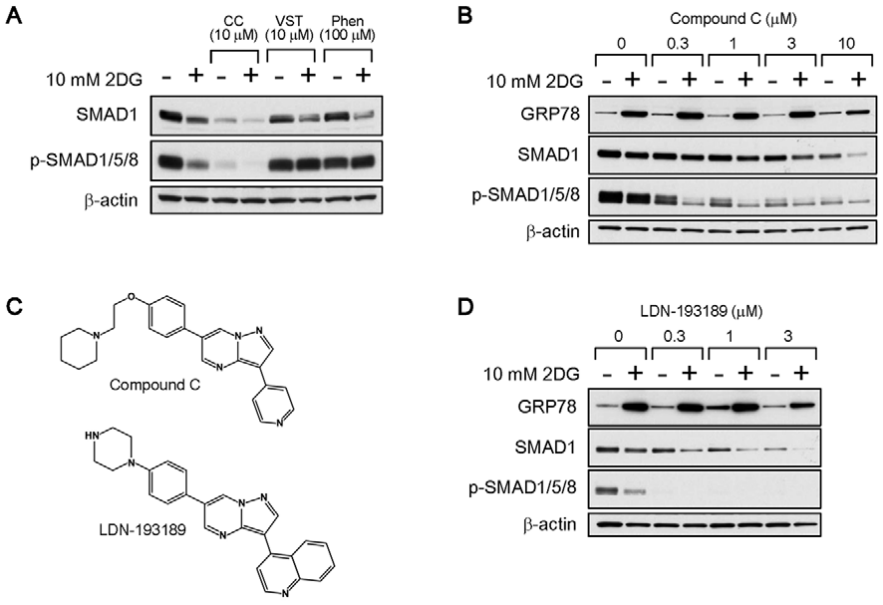
Effects of the inhibition of SMAD phosphorylation on GRP78 accumulation. (A) Immunoblot analysis. HT1080 cells were treated with compound C, versipelostatin or phenformin for 18 hours in the presence (+) or absence (−) of 10 mM 2DG. CC, compound C; VST, versipelostatin; Phen, phenformin. β-actin was used as a loading control. (B, D) Immunoblot analysis. HT1080 cells were treated with various concentrations of compound C (B) or LDN-193189 (D) for 18 hours in the presence (+) or absence (−) of 10 mM 2DG. β-actin was used as a loading control. (C) Structure of compound C and LDN-193189.

Compound C has been shown to block SMAD1/5/8 phosphorylation by inhibiting BMP signaling pathways [21], [24]. Indeed, we found that compound C profoundly reduced phosphorylation levels of SMAD1/5/8 as well as SMAD1 expression levels in HT1080 cells (Figure 5A). The classic UPR inhibitors versipelostatin and phenformin had no effect on SMAD phosphorylation, although 2DG had a moderate effect. A dose-dependent experiment revealed that compound C inhibited SMAD1/5/8 phosphorylation at as low as 1 µM, a concentration that did not inhibit 2DG-induced GRP78 expression (Figure 5B). LDN-193189, a compound C analog that is more selective as a BMP signaling inhibitor (Figure 5C) [24], [25], [26], also reduced SMAD1/5/8 phosphorylation more potently than compound C but did not inhibit 2DG-induced GRP78 expression (Figure 5D). Consistent with these results, SMAD1 knockdown hardly affected the GRP78 protein accumulation under 2DG stress conditions (Figure S3). These results indicated that inhibition activities of compound C were clearly different for the BMP signaling and the UPR.

### Synergy between Compound C and Classical UPR Inhibitors

**Figure 6:**
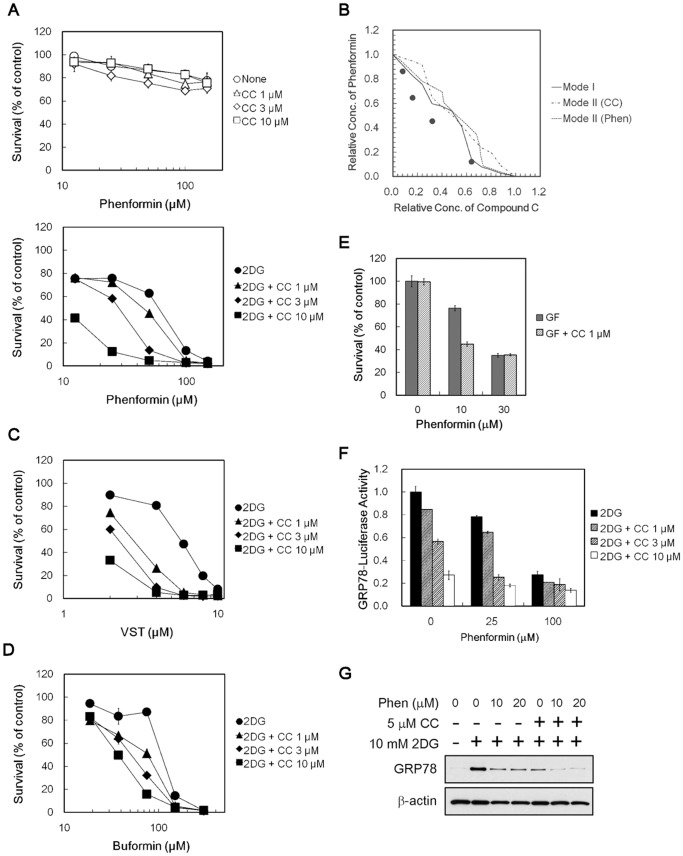
Combined effects of compound C and biguanides/versipelostatin in cancer cells during glucose deprivation. (A) MTT assay. 786-O cells were treated with compound C and phenformin under normal (*upper*) or 10 mM 2DG stress (*lower*) conditions. Results shown are the means ± SD of quadruplicate determinations. (B) Isobologram analysis for combined effects of compound C and phenformin under 2DG stress conditions. (C, D) MTT assay. 786-O cells were treated with compound C and versipelostatin (C) or buformin (D) in the presence of 10 mM 2DG. Results shown are the means ± SD of quadruplicate determinations. (E) MTT assay. HT1080 cells were treated with 1 µM compound C and 10 µM phenformin during glucose withdrawal. Results shown are the means ± SD of duplicate determinations. (F) Reporter assay. HT1080 cells were transfected with pGRP78pro160-Luc and treated with compound C and phenformin for 18 h in the presence of 10 mM 2DG. Results shown are the means ± SD of quadruplicate determinations. (G) Immunoblot analysis. HT1080 cells were treated for 6 h with various concentration of phenformin in the presence (+) or absence (−) of 10 mM 2DG and 5 µM compound C. β-actin was used as a loading control. GF, glucose-free; CC, compound C; Phen, phenformin.

Given that compound C and classic UPR inhibitors had different mode of actions on UPR inhibition, we examined the potential of their combined use. For this purpose, we predetermined the experimental conditions, where compound C exerted sublethal toxicity during glucose deprivation. Under these conditions, compound C showed similar weak cytotoxicity in the presence or absence of 2DG (Figure S4). We found that treating 786-O cells with combination compound C and phenformin caused greater cytotoxicity than did either compound alone under 2DG stress conditions (Figure 6A, *lower*). Isobologram analysis revealed that this combination produced synergistic cytotoxicity under 2DG stress conditions (Figure 6B). Importantly, the compound C–phenformin combination did not enhance cytotoxicity under normal growth conditions (Figure 6A, *upper*). Enhanced cytotoxicity was seen when compound C was used in combination with versipelostatin and buformin in 2DG-stressed 786-O cells (Figure 6C, D) and in combination with phenformin in HT1080 cells under glucose withdrawal conditions (Figure 6E). As shown in Figure 6F and 6G, the combination of compound C and phenformin resulted in enhanced inhibition of GRP78 promoter activity and GRP78 protein accumulation under 2DG stress conditions (Figure 6F, G).

## Discussion

We have identified compound C as a novel, small-molecule inhibitor of the UPR transcription program. Similar to previously discovered inhibitors versipelostatin and biguanides, compound C’s UPR-inhibitory activity and enhanced cytotoxicity depended on glucose deprivation. However, our gene expression profiling and biochemical analysis revealed that the mode of action of compound C was not the same as versipelostatin and phenformin. The differential modes of action were further supported by synergistic interactions between compound C and the classic inhibitors on both UPR inhibition and cell death induction during glucose deprivation. We also demonstrated that UPR-inhibitory activity of compound C was dissociated with its known activities, including AMPK and BMP signaling inhibition.

In agreement with previous study, compound C inhibited AMPK phosphorylation under normal growth and glucose deprivation conditions. Whereas AMPK activation has been shown to promote metabolic changes to maintain cell proliferation and survival, recent reports indicated that AMPK also inhibited the ATP-catabolic processes and caused cell-growth arrest and/or apoptosis [27]. Thus, AMPK signaling pathway can mediate opposite, prosurvival and proapoptotic functions, depending on cell types and/or cell conditions, but the regulatory mechanism is unknown. In this context, although our present data demonstrate that AMPK is dispensable for UPR activation during glucose deprivation, it is still possible that the AMPK signaling pathway can be involved in determining cellular fate during glucose deprivation through the identified and/or unidentified mechanisms.

Apart from AMPK and BMP signaling, compound C has been shown to exert certain “off-target” biological effects, such as inhibiting vascular endothelial growth factor type II receptor and inhibiting hypoxia-inducible factor-1 activation [26], [28], [29]. It is conceivable that these multiple biological effects stem, at least in part, from existence of multiple targets for this drug. In fact, kinase inhibition profiling, using a panel consisting of over 70 recombinant protein kinases, has revealed that compound C not only inhibits AMPK but also a number of other kinases with similar or greater potencies [30]. Although it is currently unknown whether compound C–sensitive kinases can be involved in UPR regulation, the UPR is regulated by many kinases, e.g. the ER-localized stress sensor PERK [1], [3]. In this context, it would be interesting to conduct a structure-activity relation (SAR) study of compound C analogs for novel kinase inhibitors that modulate the UPR, like the previously successful SAR study of development of the selective BMP signaling inhibitor LDN-193189 [25].

It would also be interesting to search for chemicals that exert similar biological effects. For example, we recently developed a chemical genomics approach to searching glucose deprivation–selective UPR inhibitors, based on gene expression profiling with versipelostatin and biguanides [15], [31]. In fact, using this approach with the Connectivity Map system [32], we have succeeded in identifying several chemicals as UPR inhibitors. Those include an antihelminthic pyrvinium pamoate, a potassium ionophore valinomycin and the PKC inhibitor rottlerin [15], [31]. We noted that these chemicals showed remarkable similarity in their modes of action, in spite of differences in chemical structure, and have reported our discovery of the common mechanisms leading to UPR inhibition [15], [16], [17]. Similar chemical genomics approaches, as well as other informatics-based approaches [33], may be applicable to compound C and its analogs and lead to discovering a new class of small-molecule inhibitors with unique UPR inhibition mechanisms.

Considering the development of new inhibitors, it will be important to know the activity against non-tumor cells. In this report, we demonstrated that compound C, as well as known UPR inhibitors versipelostatin and phenformin, inhibited GRP78 accumulation both in tumor and non-tumor cells during glucose deprivation. The activity may provide an advantage for using these UPR inhibitors as antitumor agents, because various non-tumor normal cells assist tumor development under microenvironmental conditions, including glucose deprivation. On the other hand, there is a possibility that compound C has potential adverse effects in certain normal cells. In fact, the UPR can be a cellular adaptive mechanism to cope with wide variety of ER stress both in tumor and normal tissues. As shown, compound C reduced PERK protein in tumor and non-tumor cells under normal growth conditions, which may cause diminished potential for certain cells to resist ER stress. Nevertheless, the UPR inhibitors used in this study can be useful for cancer therapy, because they inhibit UPR activation selectively during glucose deprivation. Notably, glucose deprivation is a common feature in poorly vascularized solid tumors, but not observed in normal tissues. Thus, we believe that disrupting the UPR during glucose deprivation would be an attractive approach to cancer treatment.

Our present findings may also be useful to establish the concept that combining different types of UPR inhibitors can produce synergistic cell death. As shown, the use of a single UPR inhibitor can suppress the UPR transcription program to the extent that it sensitizes cancer cells to stress. However, it is well-known that regulation of the UPR is a complicated process that involves at least three independent signaling pathways: ATF6, IRE1, and PERK [1], [3]. In fact, the single gene targeting of ATF6, IRE1, or PERK only partially affects the UPR transcription program [6], [34]. It is likely, therefore, that redundancy of UPR regulation mechanisms could compensate for inhibition of a single signaling pathway. This notion would provide a rationale to use simultaneously different types of inhibitors to completely disrupt the UPR. The concept of the combined use is interesting, especially when considering that some clinically useful antitumor agents are capable of modulating the UPR [14], [35], [36].

In conclusion, this study shows compound C to be an attractive lead chemical to develop a molecular probe and ultimately a novel antitumor drug that targets the complex UPR regulatory network.

## Materials and Methods

### Chemicals

Compound C (6-[4-(2-Piperidin-1-yl-ethoxy)-phenyl)]-3-pyridin-4-yl-pyrazolo [1,5-a]-pyrimidine, dorsomorphin; Calbiochem, Darmstadt, Germany) and versipelostatin [14] were prepared as a stock solution of 10 mM in dimethyl sulfoxide (DMSO). Phenformin (Sigma, St. Louis, MO, USA), buformin (Wako, Osaka, Japan) and 2-deoxy-D-glucose (2DG) (Sigma) were dissolved in sterilized distilled water at stock concentrations of 1 M, 100 mM and 2 M, respectively. LDN-193189 (Wako), tunicamycin (Nacalai Tesque, Kyoto, Japan) and MG132 (Peptide Institute, Osaka, Japan) were dissolved in DMSO at stock concentrations of 10 mM, 4 mg/mL and 10 mM, respectively. All the stock solutions were stored at −20°C. DMSO represented less than 0.5% of the medium volume.

### Cell Culture and Treatments

Human fibrosarcoma HT1080 (CCL-121), renal cell carcinoma 786-O (CRL-1932) and cervical carcinoma HeLa cells (CCL-2) were obtained from the American Type Culture Collection (Manassas, VA, USA). Human fetal lung fibroblast TIG-3 cells (JCRB0506) were obtained from the Japanese Collection of Research Bioresources (Osaka, Japan). Cells were maintained in RPMI-1640 medium (Wako; for HT1080, 786-O and TIG-3 cells) or in Dulbecco’s modified Eagle’s medium (DMEM) (Wako; for HeLa cells). Both media, containing 2 mg/mL of glucose, were supplemented with 10% heat-inactivated fetal bovine serum (FBS) and 100 µg/mL of kanamycin. All cell lines were cultured at 37°C in a humidified atmosphere containing 5% CO_2_ as the normal growth condition.

To induce the UPR, we treated cells for various times under ER stress conditions by replacing the medium with glucose-free medium (glucose withdrawal) or by adding either 2DG or tunicamycin to glucose-containing medium. The glucose-free RPMI-1640 and DMEM (Invitrogen, Carlsbad, CA, USA) were supplemented with 10% heat-inactivated FBS [37]. Compound C, versipelostatin and phenformin were added at various final concentrations immediately after cells were placed in glucose-free medium or just before the chemical stressors were added to glucose-containing culture medium. In some immunoblot analyses, we treated cells with the proteasome inhibitor MG132 at 10 µM during exposure to the UPR inducers.

### Plasmids and Transfection

The pcFlag vector, the pGRP78pro160-Luc and the 7× FLAG-tagged ATF6 have been described previously [14], [38]. cDNA of the human AMPK wild-type α1 subunit was subcloned into the KpnI/EcoRI site of pcFlag plasmid vector (pAMPKα1-wt). The AMPK α1 dominant negative form (pAMPKα1-D168A), in which Asp168 was replaced with alanine [39], was generated using a QuikChange site-directed mutagenesis kit (Stratagene, La Jolla, CA, USA) according to the manufacturer’s instructions. The sequences of oligonucleotides using mutagenesis were: Fw: 5′- GAA TGC AAA GAT AGC TGC TTT TGG TCT TTC AAA C - 3′, Rv: 5′- GTT TGA AAG ACC AAA AGC AGC TAT CTT TGC ATT C - 3′. The proper construction of plasmids was confirmed by DNA sequencing. Transient transfections of plasmid DNA were performed using the Lipofectamine 2000 Reagent (Invitrogen) with antibiotic-free RPMI-1640 medium supplemented with 5% FBS, according to the manufacturer’s protocol.

### Stable XBP1-Luc/HT1080 Cell Lines

For XBP1-Luc/HT1080 cells that stably overexpressed the XBP1-Luc reporter gene [15], we generated the FLAG-tagged XBP1-Luc cloned into the pLPCX retroviral vector (Clontech, Mountain View, CA, USA). A high-titer virus stock was produced by transfecting pLPCX-XBP1-Luc into AmphoPack-293 cells (Clontech) and infecting HT1080 cells according to the manufacturer’s instructions.

### RNAi Experiments

Small interfering RNA knockdown experiments were performed with Stealth RNAi (Invitrogen). Stealth RNAi for AMPKα1 and LKB1 are as follows: PRKAA1 (HSS108454, HSS108455, HSS108456), STK11 (HSS110329, HSS110330). The Stealth RNAi negative control (Invitrogen) was used as an siRNA control. Transfections were performed using the Lipofectamine RNAiMAX transfection reagent (Invitrogen) with antibiotic-free RPMI-1640 medium supplemented with 5% FBS, according to the manufacturer’s protocol.

### Reporter Assay

A reporter assay was performed as described previously [14] (see also Text S1). Briefly, HT1080 cells were transfected with firefly luciferase–containing reporter plasmids (pGRP78pro160-Luc) and renilla luciferase–containing plasmid phRL-CMV (Promega, Fitchburg, WI, USA) as an internal control. XBP1-Luc/HT1080 cells were transfected with only phRL-CMV. Relative activity of firefly luciferase to renilla luciferase (mean ± SD of triplicate determinations) was determined using the Dual-Glo Luciferase Assay System (Promega).

### Immunoblot Analysis

Immunoblot analysis was performed as described previously [14] (see also Text S1). Briefly, equal amounts of proteins were resolved on an SDS-polyacrylamide gradient gel and transferred by electroblotting onto a nitrocellulose membrane. Membranes were probed with the indicated primary antibodies. The specific signals were visualized with a chemiluminescence detection system using appropriate secondary antibodies (PerkinElmer, Waltham, MA, USA).

### Cell Viability Assay

The 3-(4,5-dimethylthiazol-2-yl)-2,5-diphenyltetrazolium bromide (MTT) assay was performed, as described previously [14] (see also Text S1). Briefly, HeLa and 786-O cells were treated with various concentrations of compound C, versipelostatin and phenformin in the presence or absence of 10 mM 2DG or 1 µg/mL of tunicamycin as a stressor for 30 h in 96-well plates. For the combination study, 786-O cells are treated with various concentrations of UPR inhibitors in the presence or absence of 10 mM 2DG for 24 h. The medium was then replaced with fresh growth medium, and cells were cultured for a further 15 h. Subsequently, MTT (Sigma) was added to the culture medium, and the absorbance of each well was determined as described previously [40]. For the viability assay under glucose-withdrawal conditions, HT1080 cells were treated with various concentrations of compound C and phenformin in 12-well plates in the presence or absence of glucose for 18 h, seeded in 96-well plates with growth medium, and then cultured for a further 48 h before MTT was added. Relative cell survival (mean ± SD of quadruplicate determinations) was calculated by setting each control absorbance from untreated cells as 100%. The effects of drug combinations at concentrations producing 80% cell growth inhibition (IC_80_) were analyzed using the isobologram method [41].

### Microarray Analysis

Microarray analysis was done according to standard Affymetrix protocols using GeneChip Human Genome U133 Plus 2.0 arrays (see also Text S1). The microarray data sets were deposited in the national Center for Biotechnology Information Gene Expression Omnibus under the series accession No. GSE32911.

### PCR Analysis

Detection of splicing variants and quantitative real-time PCR of endogenous XBP1 mRNA were performed as described previously [14], [15] (see also Text S1). Briefly, HT1080 cells were seeded in 6-well plates and treated with compound C and versipelostatin in the presence or absence of 10 mM 2DG for 18 h. Total RNA was isolated from the cells using the RNeasy Mini Kit (Qiagen, Hilden, Germany) and converted to cDNA with SuperScript II reverse transcriptase (Invitrogen). To detect the XBP1 mRNA splicing valiant, each cDNA was amplified using specific primer pairs with Taq DNA polymerase (Roche) and then each PCR product was analyzed with the 2100 Bioanalyzer (Agilent Technologies, Santa Clara, CA, USA). The quantitative real-time PCR was performed using FAM-labeled D-LUX primer sets (Invitrogen) and analyzed with the ABI PRISM 7700 (Applied Biosystems, Foster City, CA, USA).

### AMPK Kinase Assay (ELISA Assay)

HT1080 cells were seeded in 24-well plates (2×10^4^ cells per well) and treated with compound C in the presence or absence of glucose or 10 mM 2DG for 2 h. HT1080 cells that overexpressed the wild-type and dominant negative AMPKα1 were prepared by transfecting plasmid DNA (pAMPKα1-wt, pAMPKα1-D168A and pcFlag as a control) in 6-well plates, seeding in 24-well plate and treating with UPR inhibitors. Cells were lysed with cell lysis buffer (20 mM Tris-HCl, pH 7.5, 250 mM NaCl, 10% glycerol, 0.5% NP-40, 1 mM EDTA, 1 mM EGTA, 0.2 mM PMSF, 1 µg/mL pepstatin, 0.5 µg/mL leupeptin, 5 mM NaF, 2 mM Na_3_Vo_4_, 2 mM β-glycerophosphate, 1 mM DTT). Relative AMPK kinase activity (mean ± SD of duplicate determinations) to control sample (vehicle or pcFlag under normal growth conditions) was determined using the CycLex AMPK kinase assay kit (CycLex Co., Ltd., Nagano, Japan) according to the manufacturer’s instructions.

## Supporting Information

Figure S1 **Heat map of significantly expressed 8,778 probe sets in microarray analysis.** 8,778 probe sets (*X* axis) sorted by cluster analysis displayed with 7 samples (*Y* axis). HT1080 cells were cultured with 10 µM compound C, 10 µM versipelostatin and 100 µM phenformin for 18 h in the presence or absence of 10 mM 2DG. The log ratio for each gene was calculated by setting the expression level in appropriate control samples (non–drug-treated cells) as 0 (Log_2_ 1). Details of the experimental conditions are provided as Table S1. 2DG, 2-deoxy-D-glucose; CC, compound C; VST, versipelostatin; Phen, phenformin.
(TIF)

Figure S2 **Effects of compound C on AMPKα phosphorylation. Immunoblot analysis.** HT1080 cells were treated with compound C for 8 or 18 h in the presence (+) or absence (−) of 10 mM 2DG. β-actin was used as a loading control.
(TIF)

Figure S3 **Effects of SMAD1 knockdown on GRP78 accumulation.** Immunoblot analysis. HT1080 cells were transfected with SMAD1 siRNA and cultured for 18 h in the presence (+) or absence (−) of 10 mM 2DG. β-actin was used as a loading control.
(TIF)

Figure S4 **Cytotoxicity of single-treatment compound C in unstressed and 2DG-stressed 786-O cells.** MTT assay. 786-O cells were treated with compound C for 24 h under normal or 10 mM 2DG stress conditions. Results shown are the means ± SD of quadruplicate determinations.
(TIF)

Table S1 **Summary of 8 samples using microarray analysis.**
(PDF)

Table S2 **Expression level of upregulated genes (148 probe sets) in the Glucose Deprivation signature.**
(PDF)

Table S3 **Expression level of downregulated genes (98 probe sets) in the Glucose Deprivation signature.**
(PDF)

Text S1 **Supplementary Methods.**
(PDF)
