# Supplementary figures and images for: Compound C Prevents the Unfolded Protein Response during Glucose Deprivation through a Mechanism Independent of AMPK and BMP Signaling
Source: PLoS One. 2012 Sep 24;7(9):e45845. doi: 10.1371/journal.pone.0045845 (PMC3454318; doi:10.1371/journal.pone.0045845)

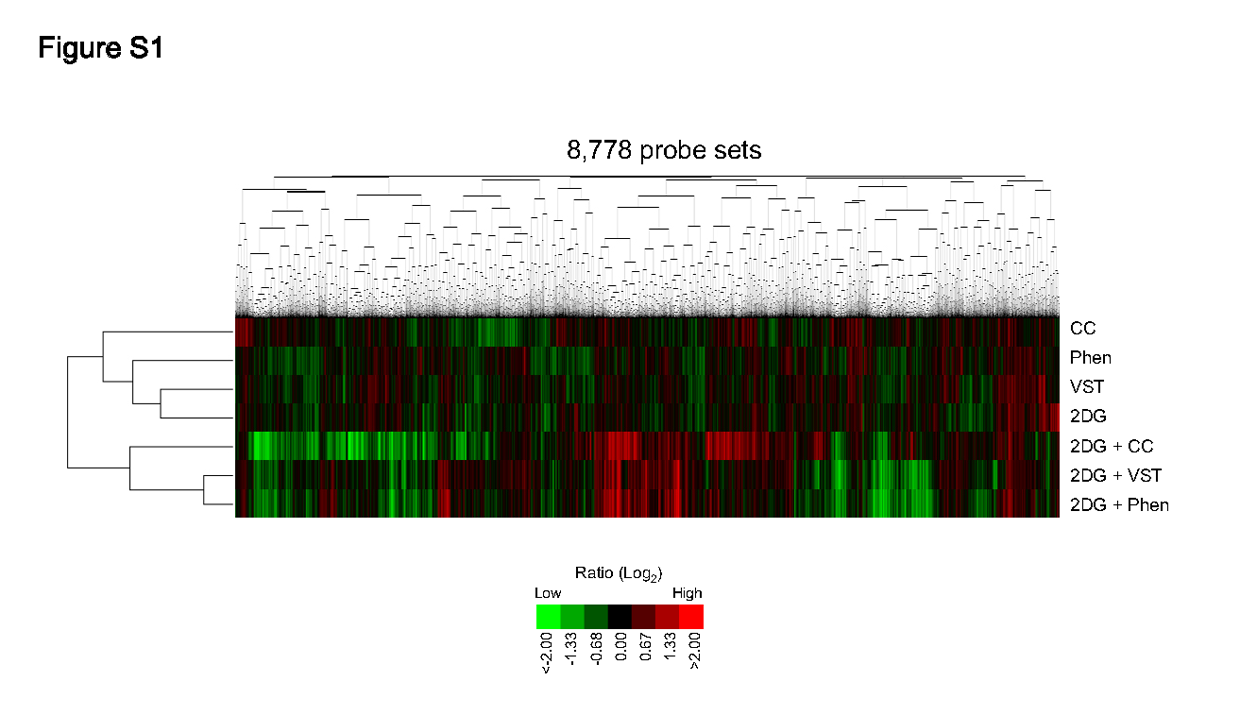

Supplement: Figure S1 — Heat map of significantly expressed 8,778 probe sets in microarray analysis. 8,778 probe sets (X axis) sorted by cluster analysis displayed with 7 samples (Y axis). HT1080 cells were cultured with 10 µM compound C, 10 µM versipelostatin and 100 µM phenformin for 18 h in the presence or absence of 10 mM 2DG. The log ratio for each gene was calculated by setting the expression level in appropriate control samples (non–drug-treated cells) as 0 (Log2 1). Details of the experimental conditions are provided as Table S1. 2DG, 2-deoxy-D-glucose; CC, compound C; VST, versipelostatin; Phen, phenformin. (TIF) [file pone.0045845.s001.tif]

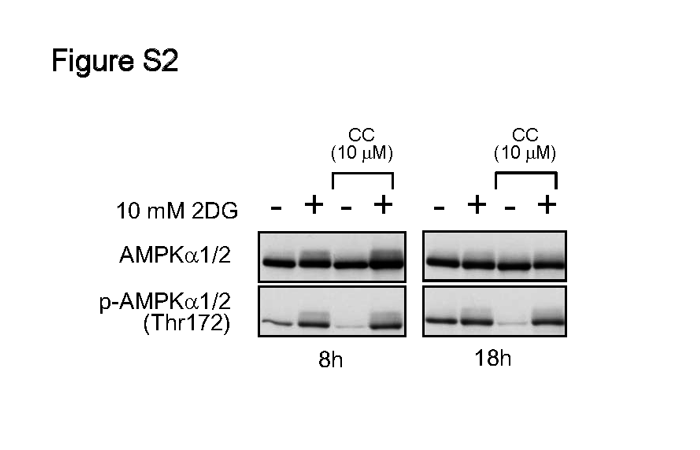

Supplement: Figure S2 — Effects of compound C on AMPKα phosphorylation. Immunoblot analysis. HT1080 cells were treated with compound C for 8 or 18 h in the presence (+) or absence (−) of 10 mM 2DG. β-actin was used as a loading control. (TIF) [file pone.0045845.s002.tif]

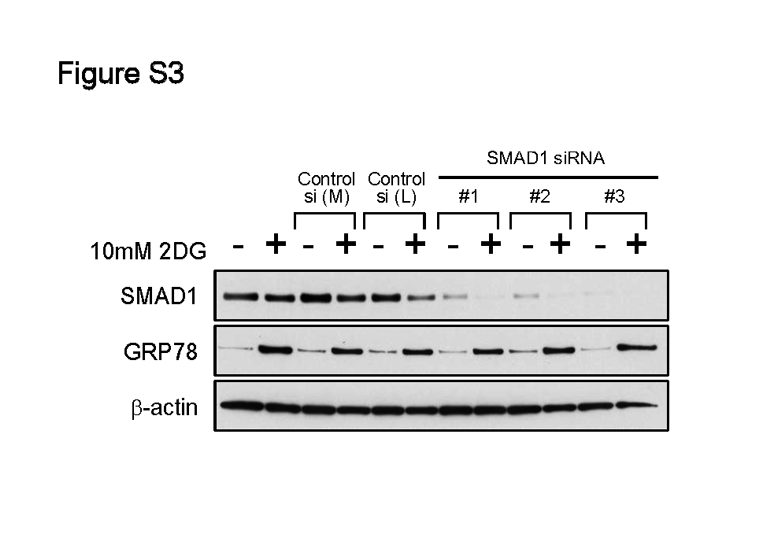

Supplement: Figure S3 — Effects of SMAD1 knockdown on GRP78 accumulation. Immunoblot analysis. HT1080 cells were transfected with SMAD1 siRNA and cultured for 18 h in the presence (+) or absence (−) of 10 mM 2DG. β-actin was used as a loading control. (TIF) [file pone.0045845.s003.tif]

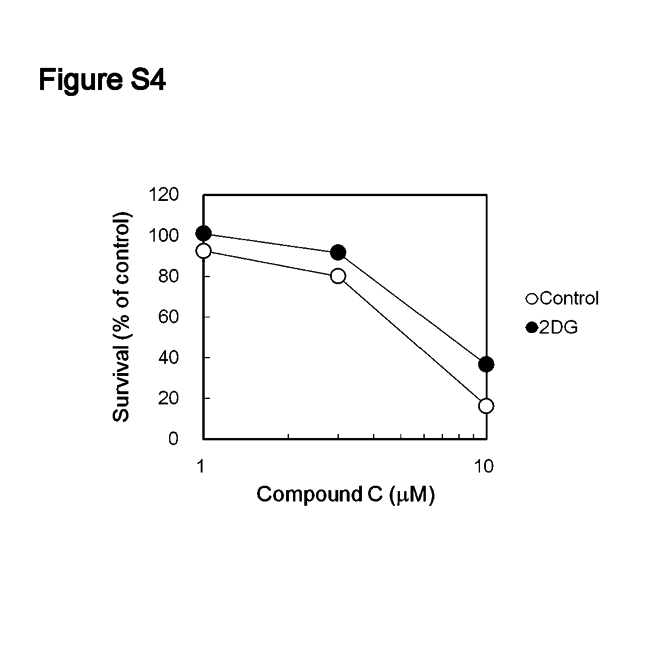

Supplement: Figure S4 — Cytotoxicity of single-treatment compound C in unstressed and 2DG-stressed 786-O cells. MTT assay. 786-O cells were treated with compound C for 24 h under normal or 10 mM 2DG stress conditions. Results shown are the means ± SD of quadruplicate determinations. (TIF) [file pone.0045845.s004.tif]
