# Supplementary material for: Compound C Prevents the Unfolded Protein Response during Glucose Deprivation through a Mechanism Independent of AMPK and BMP Signaling
Source: PLoS One. 2012 Sep 24;7(9):e45845. doi: 10.1371/journal.pone.0045845 (PMC3454318; doi:10.1371/journal.pone.0045845)
Supplement: Table S1 — Summary of 8 samples using microarray analysis. (PDF) [file pone.0045845.s005.pdf]

Table S1. Summary of 8 samples using microarray analysis

Sample Name	Cell	Compound	Concentration	Time
Cont	HT1080	–	–	18h
CC	HT1080	Compound C	10 μM	18h
VST	HT1080	VST	10 μM	18h
Phen	HT1080	Phenformin	100 μM	18h
2DG	HT1080	2DG	10 mM	18h
2DG+CC	HT1080	2DG / Compound C	10 mM / 10 μM	18h
2DG+VST	HT1080	2DG / VST	10 mM / 10 μM	18h
2DG+Phen	HT1080	2DG / Phenformin	10 mM / 100 μM	18h
