# Supplementary material for: Compound C Prevents the Unfolded Protein Response during Glucose Deprivation through a Mechanism Independent of AMPK and BMP Signaling
Source: PLoS One. 2012 Sep 24;7(9):e45845. doi: 10.1371/journal.pone.0045845 (PMC3454318; doi:10.1371/journal.pone.0045845)
Supplement: Table S2 — Expression level of upregulated genes (148 probe sets) in the Glucose Deprivation signature. (PDF) [file pone.0045845.s006.pdf]

Table S2. Expression level of upregulated genes (148 probe sets) in the Glucose Deprivation signature.

	Signal Intensity (Log_2)								Signal Log Ratio									
Probesets	Cont	CC	VST	Phen	2DG	2DG+CC	2DG+VST	2DG+Phen	Cont	CC	VST	Phen	2DG	2DG+CC	2DG+VST	2DG+Phen	Gene Symbol	Gene Title
1552639_at	3.079	2.832	3.444	3.395	4.100	3.685	3.347	3.644	0	-0.25	0.36	0.32	1.02	0.61	0.27	0.56	KLHDC7B	kelch domain containing 7B
1553055_a_at	2.447	3.509	4.259	5.065	3.643	1.900	2.053	2.123	0	1.06	1.81	2.62	1.20	-0.55	-0.39	-0.32	SLFN5	schlafen family member 5
1554148_a_at	5.698	6.411	6.510	6.124	7.445	4.351	4.117	4.938	0	0.71	0.81	0.43	1.75	-1.35	-1.58	-0.76	SLC33A1	solute carrier family 33 (acetyl-CoA transporter), member
1554462_a_at	5.613	6.426	6.721	6.689	7.377	5.633	7.724	8.436	0	0.81	1.11	1.08	1.76	0.02	2.11	2.82	DNAJB9	DnaJ (Hsp40) homolog, subfamily B, member 9
1555788_a_at	5.613	7.254	7.982	5.999	7.828	6.384	4.796	4.836	0	1.64	2.37	0.39	2.22	0.77	-0.82	-0.78	TRIB3	tribbles homolog 3 (Drosophila)
1558080_s_at	4.554	5.434	4.969	4.414	6.623	7.109	5.312	7.012	0	0.88	0.42	-0.14	2.07	2.55	0.76	2.46	DNAJC3	DnaJ (Hsp40) homolog, subfamily C, member 3
1564494_s_at	7.337	7.485	7.697	7.357	9.166	8.373	7.912	7.896	0	0.15	0.36	0.02	1.83	1.04	0.57	0.56	P4HB	prolyl 4-hydroxylase, beta polypeptide
200598_s_at	10.917	10.245	11.085	10.753	12.914	11.509	11.264	12.472	0	-0.67	0.17	-0.16	2.00	0.59	0.35	1.55	HSP90B1	heat shock protein 90kDa beta (Grp94), member 1
200599_s_at	11.531	10.888	11.540	11.443	12.992	12.038	11.812	12.838	0	-0.64	0.01	-0.09	1.46	0.51	0.28	1.31	HSP90B1	heat shock protein 90kDa beta (Grp94), member 1
200656_s_at	8.321	8.311	8.591	8.346	9.787	8.915	8.548	8.610	0	-0.01	0.27	0.02	1.47	0.59	0.23	0.29	P4HB	prolyl 4-hydroxylase, beta polypeptide
200670_at	6.909	7.455	8.047	7.318	7.936	6.443	6.370	8.316	0	0.55	1.14	0.41	1.03	-0.47	-0.54	1.41	XBP1	X-box binding protein 1
200802_at	9.514	11.248	11.264	10.222	11.060	10.519	9.581	9.834	0	1.73	1.75	0.71	1.55	1.01	0.07	0.32	SARS	seryl-tRNA synthetase
200825_s_at	7.530	7.559	7.958	7.467	9.641	8.035	7.862	8.281	0	0.03	0.43	-0.06	2.11	0.51	0.33	0.75	HYOU1	hypoxia up-regulated 1
200935_at	6.048	4.767	4.426	5.441	6.787	6.449	6.243	6.700	0	-1.28	-1.62	-0.61	0.74	0.40	0.20	0.65	CALR	calreticulin
200945_s_at	9.561	9.625	9.812	10.097	10.237	10.326	10.028	9.907	0	0.06	0.25	0.54	0.68	0.76	0.47	0.35	SEC31A	SEC31 homolog A (S. cerevisiae)
201000_at	8.943	9.626	9.888	9.143	9.985	7.644	8.420	8.714	0	0.68	0.94	0.20	1.04	-1.30	-0.52	-0.23	AARS	alanyl-tRNA synthetase
201983_s_at	7.752	7.745	7.953	7.854	8.548	6.710	8.048	8.024	0	-0.01	0.20	0.10	0.80	-1.04	0.30	0.27	EGFR	epidermal growth factor receptor (erythroblastic leukemia viral (v-erb-b) oncogene homolog, avian)
201984_s_at	6.885	6.291	8.344	7.668	7.903	6.299	7.142	7.821	0	-0.59	1.46	0.78	1.02	-0.59	0.26	0.94	EGFR	epidermal growth factor receptor (erythroblastic leukemia viral (v-erb-b) oncogene homolog, avian)
202061_s_at	7.797	8.196	8.599	8.060	9.376	8.337	6.954	7.930	0	0.40	0.80	0.26	1.58	0.54	-0.84	0.13	SEL1L	sel-1 suppressor of lin-12-like (C. elegans)
202062_s_at	5.455	5.975	5.731	5.727	7.185	6.131	5.079	6.740	0	0.52	0.28	0.27	1.73	0.68	-0.38	1.29	SEL1L	sel-1 suppressor of lin-12-like (C. elegans)
202064_s_at	5.331	5.388	5.292	5.235	7.129	5.692	5.361	6.658	0	0.06	-0.04	-0.10	1.80	0.36	0.03	1.33	SEL1L	sel-1 suppressor of lin-12-like (C. elegans)
202129_s_at	5.495	6.968	6.574	5.818	6.469	8.291	7.151	8.003	0	1.47	1.08	0.32	0.97	2.80	1.66	2.51	RIOK3	RIO kinase 3 (yeast)
202131_s_at	6.609	8.148	7.688	7.447	7.432	9.019	7.192	7.085	0	1.54	1.08	0.84	0.82	2.41	0.58	0.48	RIOK3	RIO kinase 3 (yeast)
202558_s_at	5.613	7.621	6.893	6.000	7.045	6.402	7.434	8.592	0	2.01	1.28	0.39	1.43	0.79	1.82	2.98	HSPA13	heat shock protein 70kDa family, member 13
202655_at	9.764	9.279	9.149	9.767	11.005	10.731	9.796	11.403	0	-0.48	-0.61	0.00	1.24	0.97	0.03	1.64	MANF	mesencephalic astrocyte-derived neurotrophic factor
202719_s_at	6.529	7.907	8.459	7.689	8.247	9.933	6.500	7.664	0	1.38	1.93	1.16	1.72	3.40	-0.03	1.14	TES	testis derived transcript (3 LIM domains)
202720_at	7.836	9.207	9.502	8.913	9.422	10.667	7.022	7.127	0	1.37	1.67	1.08	1.59	2.83	-0.81	-0.71	TES	testis derived transcript (3 LIM domains)
202721_s_at	6.686	6.618	7.739	6.940	7.157	6.591	6.335	6.728	0	-0.07	1.05	0.25	0.47	-0.10	-0.35	0.04	GFPT1	glutamine--fructose-6-phosphate transaminase 1
202722_s_at	7.026	7.259	8.300	7.295	7.557	6.779	6.441	6.884	0	0.23	1.27	0.27	0.53	-0.25	-0.58	-0.14	GFPT1	glutamine--fructose-6-phosphate transaminase 1
202842_s_at	6.454	7.248	7.813	7.597	8.432	6.643	8.601	9.202	0	0.79	1.36	1.14	1.98	0.19	2.15	2.75	DNAJB9	DnaJ (Hsp40) homolog, subfamily B, member 9
202843_at	3.876	5.148	5.866	5.916	6.270	3.805	6.769	7.814	0	1.27	1.99	2.04	2.39	-0.07	2.89	3.94	DNAJB9	DnaJ (Hsp40) homolog, subfamily B, member 9
203164_at	8.319	8.777	8.211	8.161	9.511	2.704	6.115	5.559	0	0.46	-0.11	-0.16	1.19	-5.62	-2.20	-2.76	SLC33A1	solute carrier family 33 (acetyl-CoA transporter), member
203165_s_at	5.609	5.704	6.276	6.111	7.204	2.826	4.061	3.243	0	0.10	0.67	0.50	1.59	-2.78	-1.55	-2.37	SLC33A1	solute carrier family 33 (acetyl-CoA transporter), member
203252_at	7.190	7.348	6.729	7.669	8.654	9.482	7.246	7.950	0	0.16	-0.46	0.48	1.46	2.29	0.06	0.76	CDK2AP2	cyclin-dependent kinase 2 associated protein 2
203279_at	7.239	7.469	7.564	7.195	8.157	7.025	6.427	6.073	0	0.23	0.32	-0.04	0.92	-0.21	-0.81	-1.17	EDEM1	ER degradation enhancer, mannosidase alpha-like 1
203455_s_at	7.780	10.153	9.616	9.229	8.312	10.644	10.759	10.734	0	2.37	1.84	1.45	0.53	2.86	2.98	2.95	SAT1	spermidine/spermine N1-acetyltransferase 1
203675_at	7.071	6.932	7.543	7.419	9.722	7.517	7.821	7.472	0	-0.14	0.47	0.35	2.65	0.45	0.75	0.40	NUCB2	nucleobindin 2
203827_at	5.533	6.898	7.228	6.686	7.773	4.832	5.215	4.714	0	1.36	1.69	1.15	2.24	-0.70	-0.32	-0.82	WIPI1	WD repeat domain, phosphoinositide interacting 1
203935_at	7.679	7.852	8.684	8.207	8.500	7.576	7.340	7.996	0	0.17	1.01	0.53	0.82	-0.10	-0.34	0.32	ACVR1	activin A receptor, type I
204203_at	6.911	7.761	8.879	7.892	8.439	9.782	8.921	9.409	0	0.85	1.97	0.98	1.53	2.87	2.01	2.50	CEBPG	CCAAT/enhancer binding protein (C/EBP), gamma
207668_x_at	10.296	10.139	10.052	10.235	11.622	11.173	10.499	10.935	0	-0.16	-0.24	-0.06	1.33	0.88	0.20	0.64	PDIA6	protein disulfide isomerase family A, member 6
208499_s_at	6.108	6.423	6.451	5.776	8.422	10.319	7.672	8.672	0	0.32	0.34	-0.33	2.31	4.21	1.56	2.56	DNAJC3	DnaJ (Hsp40) homolog, subfamily C, member 3
208612_at	10.636	10.300	10.521	10.559	12.272	11.405	10.887	11.140	0	-0.34	-0.11	-0.08	1.64	0.77	0.25	0.50	PDIA3	protein disulfide isomerase family A, member 3
208613_s_at	7.450	7.994	9.014	8.467	8.774	8.223	7.700	7.742	0	0.54	1.56	1.02	1.32	0.77	0.25	0.29	FLNB	filamin B, beta
208639_x_at	10.353	10.272	10.144	10.279	11.750	11.288	10.541	11.008	0	-0.08	-0.21	-0.07	1.40	0.93	0.19	0.65	PDIA6	protein disulfide isomerase family A, member 6
208658_at	7.449	6.775	6.877	7.081	9.308	7.626	7.609	7.423	0	-0.67	-0.57	-0.37	1.86	0.18	0.16	-0.03	PDIA4	protein disulfide isomerase family A, member 4
208763_s_at	5.452	8.893	7.119	9.843	7.161	9.608	5.690	7.414	0	3.44	1.67	4.39	1.71	4.16	0.24	1.96	TSC22D3	TSC22 domain family, member 3
208959_s_at	7.978	8.347	8.203	8.005	9.481	8.288	8.551	9.109	0	0.37	0.22	0.03	1.50	0.31	0.57	1.13	ERP44	endoplasmic reticulum protein 44
209099_x_at	6.880	8.462	8.565	8.232	7.948	7.591	6.809	8.058	0	1.58	1.68	1.35	1.07	0.71	-0.07	1.18	JAG1	jagged 1 (Alagille syndrome)
209238_at	7.008	6.914	6.641	6.496	8.040	9.257	6.432	7.823	0	-0.09	-0.37	-0.51	1.03	2.25	-0.58	0.82	STX3	syntaxin 3
209270_at	10.846	10.853	10.744	10.769	12.601	11.294	10.411	10.742	0	0.01	-0.10	-0.08	1.75	0.45	-0.43	-0.10	LAMB3	laminin, beta 3
209383_at	6.326	8.422	9.298	7.529	8.834	6.768	8.718	9.797	0	2.10	2.97	1.20	2.51	0.44	2.39	3.47	DDIT3	DNA-damage-inducible transcript 3
209435_s_at	7.659	8.409	9.139	8.802	8.651	7.145	7.534	8.564	0	0.75	1.48	1.14	0.99	-0.51	-0.12	0.91	ARHGEF2	Rho/Rac guanine nucleotide exchange factor (GEF) 2
209610_s_at	3.110	5.420	4.832	4.390	5.611	3.138	2.816	3.362	0	2.31	1.72	1.28	2.50	0.03	-0.29	0.25	SLC1A4	solute carrier family 1 (glutamate/neutral amino acid transporter), member 4
209682_at	5.642	6.575	6.641	6.744	6.837	7.143	4.073	5.429	0	0.93	1.00	1.10	1.19	1.50	-1.57	-0.21	CBLB	Cas-Br-M (murine) ecotropic retroviral transforming
209743_s_at	4.483	5.174	5.205	5.116	5.421	5.031	5.382	5.959	0	0.69	0.72	0.63	0.94	0.55	0.90	1.48	ITCH	itchy E3 ubiquitin protein ligase homolog (mouse)
209744_x_at	6.769	7.474	7.534	7.511	7.745	7.187	7.673	8.296	0	0.70	0.76	0.74	0.98	0.42	0.90	1.53	ITCH	itchy E3 ubiquitin protein ligase homolog (mouse)
210592_s_at	7.665	10.242	9.767	8.811	8.372	10.948	10.907	11.135	0	2.58	2.10	1.15	0.71	3.28	3.24	3.47	SAT1	spermidine/spermine N1-acetyltransferase 1
210970_s_at	7.032	7.943	7.933	7.582	7.604	5.213	6.708	6.452	0	0.91	0.90	0.55	0.57	-1.82	-0.32	-0.58	IBTK	inhibitor of Bruton agammaglobulinemia tyrosine kinase
210999_s_at	5.793	6.686	7.742	6.427	6.664	8.679	5.964	7.513	0	0.89	1.95	0.63	0.87	2.89	0.17	1.72	GRB10	growth factor receptor-bound protein 10

Table S2. (continued)

	Signal Intensity (Log_2)								Signal Log Ratio									
Probesets	Cont	CC	VST	Phen	2DG	2DG+CC	2DG+VST	2DG+Phen	Cont	CC	VST	Phen	2DG	2DG+CC	2DG+VST	2DG+Phen	Gene Symbol	Gene Title
211048_s_at	9.060	8.564	8.938	9.214	11.010	8.945	9.406	9.494	0	-0.50	-0.12	0.15	1.95	-0.12	0.35	0.43	PDIA4	protein disulfide isomerase family A, member 4
211936_at	11.187	10.584	11.550	11.281	12.894	13.417	12.332	13.411	0	-0.60	0.36	0.09	1.71	2.23	1.15	2.22	HSPA5	heat shock 70kDa protein 5 (glucose-regulated protein,
212110_at	9.966	10.028	10.226	9.737	10.858	9.570	9.673	10.069	0	0.06	0.26	-0.23	0.89	-0.40	-0.29	0.10	SLC39A14	solute carrier family 39 (zinc transporter), member 14
212810_s_at	2.824	4.396	3.224	3.086	4.687	3.297	3.263	3.039	0	1.57	0.40	0.26	1.86	0.47	0.44	0.22	SLC1A4	solute carrier family 1 (glutamate/neutral amino acid transporter), member 4
212811_x_at	4.759	5.908	5.898	5.407	6.835	4.294	4.755	4.559	0	1.15	1.14	0.65	2.08	-0.46	0.00	-0.20	SLC1A4	solute carrier family 1 (glutamate/neutral amino acid transporter), member 4
212902_at	6.051	6.245	7.116	6.693	7.038	7.640	6.959	7.053	0	0.19	1.07	0.64	0.99	1.59	0.91	1.00	SEC24A	SEC24 family, member A (S. cerevisiae)
213532_at	7.837	8.445	8.808	7.972	8.714	9.113	7.431	7.812	0	0.61	0.97	0.14	0.88	1.28	-0.41	-0.02	ADAM17	ADAM metallopeptidase domain 17
213836_s_at	5.884	7.310	7.398	7.348	8.203	4.991	5.772	5.153	0	1.43	1.51	1.46	2.32	-0.89	-0.11	-0.73	WIPI1	WD repeat domain, phosphoinositide interacting 1
213988_s_at	6.654	9.201	8.803	8.107	7.519	10.125	10.183	10.551	0	2.55	2.15	1.45	0.86	3.47	3.53	3.90	SAT1	spermidine/spermine N1-acetyltransferase 1
214316_x_at	6.133	5.218	5.750	6.128	6.971	6.870	6.767	6.947	0	-0.91	-0.38	0.00	0.84	0.74	0.63	0.81	---	---
214658_at	8.220	8.150	8.573	8.366	8.736	7.165	8.406	8.558	0	-0.07	0.35	0.15	0.52	-1.05	0.19	0.34	TMED7	transmembrane emp24 protein transport domain
216268_s_at	6.674	8.529	8.603	8.291	7.878	7.530	6.427	8.046	0	1.85	1.93	1.62	1.20	0.86	-0.25	1.37	JAG1	jagged 1 (Alagille syndrome)
216449_x_at	10.391	9.851	10.439	10.318	12.331	11.082	10.846	12.086	0	-0.54	0.05	-0.07	1.94	0.69	0.46	1.70	HSP90B1	heat shock protein 90kDa beta (Grp94), member 1
216640_s_at	9.265	9.062	8.934	9.231	10.764	10.341	9.552	9.879	0	-0.20	-0.33	-0.03	1.50	1.08	0.29	0.61	PDIA6	protein disulfide isomerase family A, member 6
216985_s_at	3.753	3.495	3.302	3.173	4.694	6.530	4.091	4.941	0	-0.26	-0.45	-0.58	0.94	2.78	0.34	1.19	STX3	syntaxin 3
217168_s_at	9.163	9.665	10.056	9.538	10.916	11.123	7.581	11.262	0	0.50	0.89	0.38	1.75	1.96	-1.58	2.10	HERPUD1	homocysteine-inducible, endoplasmic reticulum stress-inducible, ubiquitin-like domain member 1
217823_s_at	7.222	7.414	7.413	7.630	8.445	7.450	6.635	6.946	0	0.19	0.19	0.41	1.22	0.23	-0.59	-0.28	UBE2J1	ubiquitin-conjugating enzyme E2, J1 (UBC6 homolog,
217826_s_at	6.995	7.104	7.196	7.156	8.048	7.376	6.721	7.129	0	0.11	0.20	0.16	1.05	0.38	-0.27	0.13	UBE2J1	ubiquitin-conjugating enzyme E2, J1 (UBC6 homolog,
218050_at	8.691	9.881	9.066	8.767	8.963	9.647	9.641	9.169	0	1.19	0.38	0.08	0.27	0.96	0.95	0.48	UFM1	ubiquitin-fold modifier 1
218145_at	8.708	10.612	11.003	9.499	10.817	9.411	7.367	8.439	0	1.90	2.30	0.79	2.11	0.70	-1.34	-0.27	TRIB3	tribbles homolog 3 (Drosophila)
218358_at	8.229	7.782	7.363	7.968	10.232	8.818	8.226	8.704	0	-0.45	-0.87	-0.26	2.00	0.59	0.00	0.47	CRELD2	cysteine-rich with EGF-like domains 2
218615_s_at	6.137	6.445	6.237	5.779	6.744	7.034	5.050	5.770	0	0.31	0.10	-0.36	0.61	0.90	-1.09	-0.37	TMEM39A	transmembrane protein 39A
218681_s_at	7.737	7.250	6.572	6.397	9.539	8.819	7.485	8.965	0	-0.49	-1.16	-1.34	1.80	1.08	-0.25	1.23	SDF2L1	stromal cell-derived factor 2-like 1
218696_at	6.367	6.379	6.053	6.503	6.857	8.429	2.936	4.830	0	0.01	-0.31	0.14	0.49	2.06	-3.43	-1.54	EIF2AK3	eukaryotic translation initiation factor 2-alpha kinase 3
218871_x_at	6.878	7.842	7.557	7.353	8.154	8.061	7.796	8.175	0	0.96	0.68	0.47	1.28	1.18	0.92	1.30	CSGALNACT2	chondroitin sulfate N-acetylgalactosaminyltransferase 2
218972_at	6.161	6.133	6.997	6.658	8.742	5.998	5.893	5.755	0	-0.03	0.84	0.50	2.58	-0.16	-0.27	-0.41	TTC17	tetratricopeptide repeat domain 17
219600_s_at	6.935	7.055	6.410	7.455	8.599	6.088	6.827	8.041	0	0.12	-0.52	0.52	1.66	-0.85	-0.11	1.11	TMEM50B	transmembrane protein 50B
219910_at	4.136	4.948	4.077	4.170	4.966	4.417	3.936	3.993	0	0.81	-0.06	0.03	0.83	0.28	-0.20	-0.14	FICD	FIC domain containing
220012_at	2.125	2.478	2.828	2.797	3.943	2.632	2.377	3.462	0	0.35	0.70	0.67	1.82	0.51	0.25	1.34	ERO1LB	ERO1-like beta (S. cerevisiae)
220038_at	5.485	5.296	4.350	5.102	5.757	2.888	3.922	3.545	0	-0.19	-1.13	-0.38	0.27	-2.60	-1.56	-1.94	C8orf44 /// SGK3	chromosome 8 open reading frame 44 /// serum/glucocorticoid regulated kinase family, member 3
220606_s_at	6.410	6.613	7.960	7.136	7.434	5.251	6.773	7.253	0	0.20	1.55	0.73	1.02	-1.16	0.36	0.84	C17orf48	chromosome 17 open reading frame 48
221050_s_at	5.105	5.452	6.637	5.356	6.421	2.893	4.316	4.436	0	0.35	1.53	0.25	1.32	-2.21	-0.79	-0.67	GTPBP2	GTP binding protein 2
221156_x_at	2.779	3.652	3.773	3.468	5.291	2.337	3.430	3.473	0	0.87	0.99	0.69	2.51	-0.44	0.65	0.69	CCPG1	cell cycle progression 1
221511_x_at	4.645	6.067	6.169	5.412	6.280	4.086	4.384	4.436	0	1.42	1.52	0.77	1.63	-0.56	-0.26	-0.21	CCPG1	cell cycle progression 1
221553_at	7.111	6.770	6.944	7.238	8.146	7.103	7.238	6.742	0	-0.34	-0.17	0.13	1.04	-0.01	0.13	-0.37	MAGT1	magnesium transporter 1
221577_x_at	7.990	11.058	11.705	10.091	10.056	10.374	11.097	10.582	0	3.07	3.72	2.10	2.07	2.38	3.11	2.59	GDF15 /// LOC100292463	growth differentiation factor 15 /// similar to growth differentiation factor 15
221781_s_at	6.347	6.563	6.971	6.539	7.924	6.546	6.557	6.499	0	0.22	0.62	0.19	1.58	0.20	0.21	0.15	DNAJC10	DnaJ (Hsp40) homolog, subfamily C, member 10
221782_at	6.965	7.311	7.714	7.321	8.585	7.273	7.747	7.294	0	0.35	0.75	0.36	1.62	0.31	0.78	0.33	DNAJC10	DnaJ (Hsp40) homolog, subfamily C, member 10
221865_at	5.750	7.333	7.157	6.014	6.746	7.590	5.432	6.185	0	1.58	1.41	0.26	1.00	1.84	-0.32	0.44	C9orf91	chromosome 9 open reading frame 91
221985_at	3.978	5.207	6.110	5.850	6.574	5.498	4.597	6.692	0	1.23	2.13	1.87	2.60	1.52	0.62	2.71	KLHL24	kelch-like 24 (Drosophila)
222235_s_at	6.977	8.123	7.685	7.389	8.463	8.091	7.950	7.965	0	1.15	0.71	0.41	1.49	1.11	0.97	0.99	CSGALNACT2 /// LOC644504	chondroitin sulfate N-acetylgalactosaminyltransferase 2 /// novel protein similar to chondroitin sulfate GalNAcT-2
222435_s_at	6.627	6.938	7.051	6.983	8.037	7.748	6.653	7.163	0	0.31	0.42	0.36	1.41	1.12	0.03	0.54	UBE2J1	ubiquitin-conjugating enzyme E2, J1 (UBC6 homolog,
222632_s_at	6.554	7.218	7.320	6.848	8.392	7.292	7.200	7.052	0	0.66	0.77	0.29	1.84	0.74	0.65	0.50	LZTFL1	leucine zipper transcription factor-like 1
222690_s_at	6.788	6.832	7.289	7.007	7.628	8.487	6.686	7.870	0	0.04	0.50	0.22	0.84	1.70	-0.10	1.08	TMEM39A	transmembrane protein 39A
222728_s_at	9.699	10.780	10.820	10.665	10.817	10.925	10.961	11.856	0	1.08	1.12	0.97	1.12	1.23	1.26	2.16	TAF1D	TATA box binding protein (TBP)-associated factor, RNA polymerase I, D, 41kDa
222907_x_at	3.745	4.055	3.062	4.222	5.316	2.464	3.687	3.610	0	0.31	-0.68	0.48	1.57	-1.28	-0.06	-0.14	TMEM50B	transmembrane protein 50B
223054_at	9.653	9.552	9.208	9.034	11.526	11.102	9.846	10.832	0	-0.10	-0.44	-0.62	1.87	1.45	0.19	1.18	DNAJB11	DnaJ (Hsp40) homolog, subfamily B, member 11
223058_at	5.472	6.090	6.263	5.366	6.262	7.215	4.892	5.880	0	0.62	0.79	-0.11	0.79	1.74	-0.58	0.41	FAM107B	family with sequence similarity 107, member B
223059_s_at	5.246	6.020	6.304	5.552	6.447	7.591	5.301	6.110	0	0.77	1.06	0.31	1.20	2.35	0.06	0.86	FAM107B	family with sequence similarity 107, member B
223070_at	10.200	9.924	9.980	10.307	10.579	10.139	10.666	11.134	0	-0.28	-0.22	0.11	0.38	-0.06	0.47	0.93	SELK	selenoprotein K
223209_s_at	9.631	9.883	9.613	9.445	10.396	10.873	9.978	10.168	0	0.25	-0.02	-0.19	0.76	1.24	0.35	0.54	SELS	selenoprotein S
223299_at	7.696	8.227	7.646	7.544	9.478	8.211	8.222	7.864	0	0.53	-0.05	-0.15	1.78	0.51	0.53	0.17	SEC11C	SEC11 homolog C (S. cerevisiae)
223568_s_at	6.429	6.419	6.668	6.540	7.866	6.538	6.748	6.120	0	-0.01	0.24	0.11	1.44	0.11	0.32	-0.31	PPAPDC1B	phosphatidic acid phosphatase type 2 domain containing
223569_at	5.861	6.148	6.061	5.946	6.987	6.506	5.876	5.553	0	0.29	0.20	0.08	1.13	0.65	0.01	-0.31	PPAPDC1B	phosphatidic acid phosphatase type 2 domain containing
223789_s_at	2.848	2.911	3.498	3.060	3.356	3.165	3.152	3.197	0	0.06	0.65	0.21	0.51	0.32	0.30	0.35	GTPBP2	GTP binding protein 2

Table S2. (continued)

	Signal Intensity (Log$_2$)								Signal Log Ratio									
Probesets	Cont	CC	VST	Phen	2DG	2DG+CC	2DG+VST	2DG+Phen	Cont	CC	VST	Phen	2DG	2DG+CC	2DG+VST	2DG+Phen	Gene Symbol	Gene Title
224741_x_at	11.433	12.748	12.744	12.187	12.644	12.284	12.051	12.613	0	1.31	1.31	0.75	1.21	0.85	0.62	1.18	GAS5	growth arrest-specific 5 (non-protein coding)
224841_x_at	11.256	12.588	12.582	12.112	12.494	12.115	11.922	12.442	0	1.33	1.33	0.86	1.24	0.86	0.67	1.19	GAS5	growth arrest-specific 5 (non-protein coding)
224915_x_at	11.704	13.099	12.748	12.429	12.979	12.996	12.466	12.984	0	1.40	1.04	0.73	1.27	1.29	0.76	1.28	C20orf199	chromosome 20 open reading frame 199
224999_at	7.551	7.501	7.302	7.418	8.128	5.689	7.664	7.468	0	-0.05	-0.25	-0.13	0.58	-1.86	0.11	-0.08	---	---
225090_at	5.626	5.968	5.849	5.529	6.778	5.487	5.260	5.335	0	0.34	0.22	-0.10	1.15	-0.14	-0.37	-0.29	SYVN1	synovial apoptosis inhibitor 1, synoviolin
225284_at	8.499	8.867	8.513	8.239	9.859	9.981	8.514	9.610	0	0.37	0.01	-0.26	1.36	1.48	0.01	1.11	DNAJC3	DnaJ (Hsp40) homolog, subfamily C, member 3
225956_at	3.854	5.260	6.026	6.364	6.449	5.741	4.722	6.662	0	1.41	2.17	2.51	2.60	1.89	0.87	2.81	C5orf41	chromosome 5 open reading frame 41
225957_at	4.572	5.679	6.810	6.766	7.095	6.413	5.108	6.718	0	1.11	2.24	2.19	2.52	1.84	0.54	2.15	C5orf41	chromosome 5 open reading frame 41
226150_at	7.639	7.014	7.851	7.573	8.892	7.388	8.249	8.033	0	-0.62	0.21	-0.07	1.25	-0.25	0.61	0.39	PPAPDC1B	phosphatidic acid phosphatase type 2 domain containing
226321_at	7.429	7.313	7.105	7.502	7.882	3.475	4.639	4.995	0	-0.12	-0.32	0.07	0.45	-3.95	-2.79	-2.43	LYSMD3	LysM, putative peptidoglycan-binding, domain containing
226384_at	5.743	6.423	6.671	6.307	7.141	6.256	5.605	5.202	0	0.68	0.93	0.56	1.40	0.51	-0.14	-0.54	PPAPDC1B	phosphatidic acid phosphatase type 2 domain containing
226835_s_at	11.459	12.872	12.538	12.135	12.751	12.848	12.287	12.833	0	1.41	1.08	0.68	1.29	1.39	0.83	1.37	C20orf199	chromosome 20 open reading frame 199
226886_at	5.852	6.227	6.854	5.690	5.975	5.653	5.166	3.974	0	0.38	1.00	-0.16	0.12	-0.20	-0.69	-1.88	GFPT1	glutamine--fructose-6-phosphate transaminase 1
227027_at	5.952	6.400	7.059	5.711	6.433	6.018	4.384	3.782	0	0.45	1.11	-0.24	0.48	0.07	-1.57	-2.17	GFPT1	glutamine--fructose-6-phosphate transaminase 1
227033_at	6.247	5.468	5.944	6.078	7.950	7.043	6.884	6.748	0	-0.78	-0.30	-0.17	1.70	0.80	0.64	0.50	PDIA3	protein disulfide isomerase family A, member 3
227443_at	5.830	6.498	7.980	7.138	7.357	3.950	4.293	3.871	0	0.67	2.15	1.31	1.53	-1.88	-1.54	-1.96	C9orf150	chromosome 9 open reading frame 150
227755_at	4.723	6.737	6.240	5.142	6.294	6.595	4.585	5.073	0	2.01	1.52	0.42	1.57	1.87	-0.14	0.35	---	---
227900_at	2.618	3.528	2.853	3.341	3.079	3.836	1.578	2.405	0	0.91	0.24	0.72	0.46	1.22	-1.04	-0.21	CBLB	Cas-Br-M (murine) ecotropic retroviral transforming
228250_at	4.303	4.861	5.053	4.787	5.492	5.338	4.435	5.922	0	0.56	0.75	0.48	1.19	1.04	0.13	1.62	FNIP1	folliculin interacting protein 1
228482_at	5.656	5.879	6.124	6.216	6.977	5.423	5.229	4.883	0	0.22	0.47	0.56	1.32	-0.23	-0.43	-0.77	CDRT4	CMT1A duplicated region transcript 4
228846_at	4.463	5.354	7.073	5.709	5.930	7.785	6.955	8.042	0	0.89	2.61	1.25	1.47	3.32	2.49	3.58	MXD1	MAX dimerization protein 1
229453_at	5.815	4.886	5.145	5.025	7.305	6.896	6.526	6.330	0	-0.93	-0.67	-0.79	1.49	1.08	0.71	0.51	PDIA3	protein disulfide isomerase family A, member 3
229618_at	5.599	5.657	6.141	6.214	5.695	5.856	3.828	3.858	0	0.06	0.54	0.62	0.10	0.26	-1.77	-1.74	SNX16	sorting nexin 16
229838_at	3.619	3.655	3.493	3.809	4.995	3.651	4.166	4.045	0	0.04	-0.13	0.19	1.38	0.03	0.55	0.43	NUCB2	nucleobindin 2
230031_at	7.212	7.525	7.019	7.272	9.041	8.955	7.668	8.352	0	0.31	-0.19	0.06	1.83	1.74	0.46	1.14	HSPA5	heat shock 70kDa protein 5 (glucose-regulated protein,
230265_at	6.695	7.160	7.488	6.813	8.292	7.203	5.143	6.345	0	0.47	0.79	0.12	1.60	0.51	-1.55	-0.35	---	---
230659_at	5.837	5.987	5.829	5.721	6.563	5.813	4.088	3.791	0	0.15	-0.01	-0.12	0.73	-0.02	-1.75	-2.05	EDEM1	ER degradation enhancer, mannosidase alpha-like 1
231944_at	3.892	4.118	4.343	4.229	5.679	3.836	3.495	3.485	0	0.23	0.45	0.34	1.79	-0.06	-0.40	-0.41	ERO1LB	ERO1-like beta (S. cerevisiae)
235341_at	4.322	4.353	3.990	4.002	5.659	6.585	4.830	6.048	0	0.03	-0.33	-0.32	1.34	2.26	0.51	1.73	DNAJC3	DnaJ (Hsp40) homolog, subfamily C, member 3
236285_at	2.729	3.049	3.615	3.308	5.683	2.567	3.074	3.636	0	0.32	0.89	0.58	2.95	-0.16	0.34	0.91	KLHDC7B	kelch domain containing 7B
239077_at	2.860	2.751	2.838	2.539	3.366	3.415	3.740	3.959	0	-0.11	-0.02	-0.32	0.51	0.56	0.88	1.10	CSGALNACT2	chondroitin sulfate N-acetylgalactosaminyltransferase 2
240277_at	5.061	5.471	5.485	4.630	5.845	4.728	4.647	5.012	0	0.41	0.42	-0.43	0.78	-0.33	-0.41	-0.05	---	---
244841_at	6.046	6.722	6.528	5.991	6.739	8.337	6.368	7.276	0	0.68	0.48	-0.06	0.69	2.29	0.32	1.23	SEC24A	SEC24 family, member A (S. cerevisiae)
