# Supplementary material for: Compound C Prevents the Unfolded Protein Response during Glucose Deprivation through a Mechanism Independent of AMPK and BMP Signaling
Source: PLoS One. 2012 Sep 24;7(9):e45845. doi: 10.1371/journal.pone.0045845 (PMC3454318; doi:10.1371/journal.pone.0045845)
Supplement: Table S3 — Expression level of downregulated genes (98 probe sets) in the Glucose Deprivation signature. (PDF) [file pone.0045845.s007.pdf]

Table S3. Expression level of downregulated genes (98 probe sets) in the Glucose Deprivation signature.

	Signal Intensity (Log_2)								Signal Log Ratio									
Probesets	Cont	CC	VST	Phen	2DG	2DG+CC	2DG+VST	2DG+Phen	Cont	CC	VST	Phen	2DG	2DG+CC	2DG+VST	2DG+Phen	Gene Symbol	Gene Title
1552470_a_at	3.701	3.653	3.331	3.709	3.060	3.015	3.318	3.173	0	-0.05	-0.37	0.01	-0.64	-0.69	-0.38	-0.53	ABHD11	abhydrolase domain containing 11
1555679_a_at	6.485	6.589	5.666	5.772	5.685	4.434	4.452	3.479	0	0.10	-0.82	-0.71	-0.80	-2.05	-2.03	-3.01	RTN4IP1	reticulon 4 interacting protein 1
1555758_a_at	9.306	8.582	8.988	8.506	7.665	9.267	9.610	9.232	0	-0.72	-0.32	-0.80	-1.64	-0.04	0.30	-0.07	CDKN3	cyclin-dependent kinase inhibitor 3
1557487_at	7.758	5.796	6.083	6.225	6.517	6.055	6.747	5.915	0	-1.96	-1.67	-1.53	-1.24	-1.70	-1.01	-1.84	---	---
200685_at	7.774	7.443	7.055	7.386	6.622	4.480	7.249	6.908	0	-0.33	-0.72	-0.39	-1.15	-3.29	-0.53	-0.87	SFRS11	splicing factor, arginine/serine-rich 11
200988_s_at	7.146	6.358	6.182	5.750	6.038	6.344	6.988	6.102	0	-0.79	-0.96	-1.40	-1.11	-0.80	-0.16	-1.04	PSME3	proteasome (prosome, macropain) activator subunit 3 (PA28 gamma; Ki)
201038_s_at	7.916	7.532	7.066	6.929	6.946	5.435	5.806	5.370	0	-0.38	-0.85	-0.99	-0.97	-2.48	-2.11	-2.55	ANP32A	acidic (leucine-rich) nuclear phosphoprotein 32 family, member A
201129_at	9.057	8.237	8.204	8.171	7.291	7.356	8.872	9.010	0	-0.82	-0.85	-0.89	-1.77	-1.70	-0.19	-0.05	SFRS7	splicing factor, arginine/serine-rich 7, 35kDa
201316_at	8.437	8.030	7.326	7.434	7.292	7.005	8.457	7.838	0	-0.41	-1.11	-1.00	-1.15	-1.43	0.02	-0.60	PSMA2	proteasome (prosome, macropain) subunit, alpha type, 2
201890_at	10.403	9.715	9.369	9.426	8.177	8.956	10.588	10.545	0	-0.69	-1.03	-0.98	-2.23	-1.45	0.18	0.14	RRM2	ribonucleotide reductase M2
201896_s_at	8.047	6.552	7.575	6.980	6.006	5.223	7.389	6.991	0	-1.50	-0.47	-1.07	-2.04	-2.82	-0.66	-1.06	PSRC1	proline/serine-rich coiled-coil 1
201969_at	7.274	7.005	5.775	5.859	5.410	5.441	6.447	6.166	0	-0.27	-1.50	-1.42	-1.86	-1.83	-0.83	-1.11	NASP	nuclear autoantigenic sperm protein (histone-binding)
202094_at	8.391	8.026	7.228	7.377	6.884	8.302	8.227	7.225	0	-0.36	-1.16	-1.01	-1.51	-0.09	-0.16	-1.17	BIRC5	baculoviral IAP repeat-containing 5
202240_at	6.048	5.281	5.729	5.007	4.718	4.641	5.467	5.168	0	-0.77	-0.32	-1.04	-1.33	-1.41	-0.58	-0.88	PLK1	polo-like kinase 1 (Drosophila)
203145_at	8.003	7.663	7.583	7.523	7.167	6.488	8.238	8.047	0	-0.34	-0.42	-0.48	-0.84	-1.51	0.23	0.04	SPAG5	sperm associated antigen 5
203276_at	9.077	8.652	8.095	8.498	7.589	5.619	8.341	7.774	0	-0.43	-0.98	-0.58	-1.49	-3.46	-0.74	-1.30	LMNB1	lamin B1
203418_at	9.381	8.025	8.839	8.521	7.686	5.956	9.359	8.824	0	-1.36	-0.54	-0.86	-1.70	-3.43	-0.02	-0.56	CCNA2	cyclin A2
203625_x_at	9.937	8.955	8.487	8.538	8.499	8.352	8.601	8.289	0	-0.98	-1.45	-1.40	-1.44	-1.59	-1.34	-1.65	SKP2	S-phase kinase-associated protein 2 (p45)
203626_s_at	4.468	3.042	3.139	2.910	2.885	3.052	3.139	3.194	0	-1.43	-1.33	-1.56	-1.58	-1.42	-1.33	-1.27	SKP2	S-phase kinase-associated protein 2 (p45)
203744_at	8.448	7.792	7.270	7.914	7.338	5.215	7.261	6.827	0	-0.66	-1.18	-0.53	-1.11	-3.23	-1.19	-1.62	HMGB3	high-mobility group box 3
204067_at	5.872	5.455	4.698	5.459	5.123	2.737	4.718	4.078	0	-0.42	-1.17	-0.41	-0.75	-3.14	-1.15	-1.79	SUOX	sulfite oxidase
204159_at	8.378	6.681	7.016	8.480	6.535	4.459	7.618	7.096	0	-1.70	-1.36	0.10	-1.84	-3.92	-0.76	-1.28	CDKN2C	cyclin-dependent kinase inhibitor 2C (p18, inhibits CDK4)
204444_at	8.441	7.504	7.274	7.599	6.881	3.479	7.912	7.717	0	-0.94	-1.17	-0.84	-1.56	-4.96	-0.53	-0.72	KIF11	kinesin family member 11
204603_at	7.058	6.177	5.922	6.166	5.769	6.024	6.645	6.540	0	-0.88	-1.14	-0.89	-1.29	-1.03	-0.41	-0.52	EXO1	exonuclease 1
204827_s_at	4.861	3.995	4.193	3.894	3.752	3.436	3.072	3.536	0	-0.87	-0.67	-0.97	-1.11	-1.42	-1.79	-1.33	CCNF	cyclin F
205436_s_at	10.230	8.801	9.119	9.152	8.822	8.815	7.414	6.947	0	-1.43	-1.11	-1.08	-1.41	-1.41	-2.82	-3.28	H2AFX	H2A histone family, member X
205909_at	7.340	6.867	5.739	6.109	5.816	3.994	6.102	5.414	0	-0.47	-1.60	-1.23	-1.52	-3.35	-1.24	-1.93	POLE2	polymerase (DNA directed), epsilon 2 (p59 subunit)
207165_at	9.690	9.271	9.740	9.551	7.870	7.906	9.783	9.309	0	-0.42	0.05	-0.14	-1.82	-1.78	0.09	-0.38	HMMR	hyaluronan-mediated motility receptor (RHAMM)
208079_s_at	10.311	9.772	9.867	9.657	9.034	7.583	9.162	8.998	0	-0.54	-0.44	-0.65	-1.28	-2.73	-1.15	-1.31	AURKA	aurora kinase A
208937_s_at	10.393	9.068	9.539	9.957	8.402	8.212	9.816	9.371	0	-1.32	-0.85	-0.44	-1.99	-2.18	-0.58	-1.02	ID1	inhibitor of DNA binding 1, dominant negative helix-loop-helix protein
208955_at	8.668	7.761	7.837	8.359	7.235	8.553	8.979	8.280	0	-0.91	-0.83	-0.31	-1.43	-0.11	0.31	-0.39	DUT	deoxyuridine triphosphatase
209377_s_at	9.900	9.035	8.484	8.839	8.841	8.976	9.662	9.140	0	-0.87	-1.42	-1.06	-1.06	-0.92	-0.24	-0.76	HMGN3	high mobility group nucleosomal binding domain 3
209709_s_at	7.949	7.541	8.053	7.777	6.021	6.392	8.122	7.663	0	-0.41	0.10	-0.17	-1.93	-1.56	0.17	-0.29	HMMR	hyaluronan-mediated motility receptor (RHAMM)
209714_s_at	9.242	8.466	8.843	8.243	7.543	9.055	9.496	9.029	0	-0.78	-0.40	-1.00	-1.70	-0.19	0.25	-0.21	CDKN3	cyclin-dependent kinase inhibitor 3
209773_s_at	11.101	9.677	10.199	10.469	9.379	10.130	10.968	10.749	0	-1.42	-0.90	-0.63	-1.72	-0.97	-0.13	-0.35	RRM2	ribonucleotide reductase M2
209891_at	8.163	7.235	7.585	7.592	6.927	6.078	6.458	5.976	0	-0.93	-0.58	-0.57	-1.24	-2.08	-1.70	-2.19	SPC25	SPC25, NDC80 kinetochore complex component, homolog (S. cerevisiae)
210567_s_at	5.928	5.884	5.170	5.147	3.619	3.429	4.650	3.202	0	-0.04	-0.76	-0.78	-2.31	-2.50	-1.28	-2.73	SKP2	S-phase kinase-associated protein 2 (p45)
211042_x_at	9.553	8.454	8.171	8.197	8.126	7.842	8.149	7.716	0	-1.10	-1.38	-1.36	-1.43	-1.71	-1.40	-1.84	MCAM	melanoma cell adhesion molecule
211814_s_at	5.198	4.592	2.822	3.409	3.307	2.399	3.463	3.647	0	-0.61	-2.38	-1.79	-1.89	-2.80	-1.73	-1.55	CCNE2	cyclin E2
212020_s_at	6.352	5.178	5.807	5.615	4.817	4.314	5.905	5.429	0	-1.17	-0.54	-0.74	-1.54	-2.04	-0.45	-0.92	MKI67	antigen identified by monoclonal antibody Ki-67
212023_s_at	6.728	5.592	5.457	5.327	4.834	4.244	6.715	6.244	0	-1.14	-1.27	-1.40	-1.89	-2.48	-0.01	-0.48	MKI67	antigen identified by monoclonal antibody Ki-67
212170_at	7.403	6.856	6.474	6.282	5.903	4.663	6.110	5.583	0	-0.55	-0.93	-1.12	-1.50	-2.74	-1.29	-1.82	RBM12	RNA binding motif protein 12
213226_at	9.842	8.648	9.251	9.495	8.398	6.715	9.988	9.655	0	-1.19	-0.59	-0.35	-1.44	-3.13	0.15	-0.19	CCNA2	cyclin A2
213449_at	6.847	6.829	5.669	5.264	5.982	4.898	5.638	5.625	0	-0.02	-1.18	-1.58	-0.87	-1.95	-1.21	-1.22	POP1	processing of precursor 1, ribonuclease P/MRP subunit (S. cerevisiae)
213852_at	8.076	7.289	6.339	7.349	6.366	6.844	6.806	5.847	0	-0.79	-1.74	-0.73	-1.71	-1.23	-1.27	-2.23	RBM8A	RNA binding motif protein 8A
214331_at	5.852	6.067	4.086	4.153	4.520	3.790	4.428	3.551	0	0.22	-1.77	-1.70	-1.33	-2.06	-1.42	-2.30	TSFM	Ts translation elongation factor, mitochondrial
215030_at	6.662	6.480	5.857	5.664	5.493	4.951	5.955	5.309	0	-0.18	-0.80	-1.00	-1.17	-1.71	-0.71	-1.35	GRSF1	G-rich RNA sequence binding factor 1
215629_s_at	6.709	5.923	5.717	4.900	4.188	4.831	5.403	4.754	0	-0.79	-0.99	-1.81	-2.52	-1.88	-1.31	-1.95	DLEU2 /// DLEU2L	deleted in lymphocytic leukemia 2 (non-protein coding) /// deleted in lymphocytic leukemia 2-like
215731_s_at	5.992	5.861	4.380	4.916	4.593	4.666	5.038	4.981	0	-0.13	-1.61	-1.08	-1.40	-1.33	-0.95	-1.01	MPHOSPH9	M-phase phosphoprotein 9
215942_s_at	6.453	5.654	5.123	5.507	5.158	4.410	5.415	4.535	0	-0.80	-1.33	-0.95	-1.30	-2.04	-1.04	-1.92	GTSE1	G-2 and S-phase expressed 1
216870_x_at	7.256	6.279	6.086	4.857	4.734	4.916	5.557	5.130	0	-0.98	-1.17	-2.40	-2.52	-2.34	-1.70	-2.13	DLEU2	deleted in lymphocytic leukemia 2 (non-protein coding)
217645_at	6.242	5.971	4.376	5.182	4.392	6.191	6.133	5.213	0	-0.27	-1.87	-1.06	-1.85	-0.05	-0.11	-1.03	COX16	COX16 cytochrome c oxidase assembly homolog (S. cerevisiae)
217856_at	5.396	5.264	4.356	4.986	4.341	4.882	4.783	3.836	0	-0.13	-1.04	-0.41	-1.05	-0.51	-0.61	-1.56	RBM8A	RNA binding motif protein 8A
218115_at	7.022	6.163	5.499	6.457	5.981	5.695	6.997	6.272	0	-0.86	-1.52	-0.56	-1.04	-1.33	-0.03	-0.75	ASF1B	ASF1 anti-silencing function 1 homolog B (S. cerevisiae)
218726_at	8.633	7.633	8.349	8.556	7.281	6.805	8.102	8.298	0	-1.00	-0.28	-0.08	-1.35	-1.83	-0.53	-0.34	HJURP	Holliday junction recognition protein

Table S3. (continued)

	Signal Intensity (Log_2)								Signal Log Ratio									
Probesets	Cont	CC	VST	Phen	2DG	2DG+CC	2DG+VST	2DG+Phen	Cont	CC	VST	Phen	2DG	2DG+CC	2DG+VST	2DG+Phen	Gene Symbol	Gene Title
218755_at	9.004	8.103	8.529	8.139	7.105	6.360	8.422	7.987	0	-0.90	-0.48	-0.87	-1.90	-2.64	-0.58	-1.02	KIF20A	kinesin family member 20A
218866_s_at	9.151	9.009	8.314	7.957	8.163	8.851	9.299	8.937	0	-0.14	-0.84	-1.19	-0.99	-0.30	0.15	-0.21	POLR3K	polymerase (RNA) III (DNA directed) polypeptide K, 12.3
219650_at	7.665	6.523	6.164	6.848	6.522	5.833	5.764	5.768	0	-1.14	-1.50	-0.82	-1.14	-1.83	-1.90	-1.90	ERCC6L	excision repair cross-complementing rodent repair deficiency, complementation group 6-like
219918_s_at	9.675	9.073	9.340	9.289	8.854	6.327	7.576	7.228	0	-0.60	-0.33	-0.39	-0.82	-3.35	-2.10	-2.45	ASPM	asp (abnormal spindle) homolog, microcephaly associated (Drosophila)
219990_at	6.706	5.822	4.752	6.051	5.183	2.360	3.957	3.191	0	-0.88	-1.95	-0.66	-1.52	-4.35	-2.75	-3.52	E2F8	E2F transcription factor 8
220085_at	7.187	6.640	5.943	5.790	5.761	6.851	6.704	6.434	0	-0.55	-1.24	-1.40	-1.43	-0.34	-0.48	-0.75	HELLS	helicase, lymphoid-specific
220651_s_at	9.312	8.165	7.511	7.847	8.084	7.186	9.159	8.891	0	-1.15	-1.80	-1.46	-1.23	-2.13	-0.15	-0.42	MCM10	minichromosome maintenance complex component 10
221436_s_at	8.133	6.538	7.363	7.056	6.473	5.831	8.051	7.360	0	-1.60	-0.77	-1.08	-1.66	-2.30	-0.08	-0.77	CDCA3	cell division cycle associated 3
221520_s_at	8.244	7.458	7.715	7.211	7.009	6.188	7.535	7.562	0	-0.79	-0.53	-1.03	-1.24	-2.06	-0.71	-0.68	CDCA8	cell division cycle associated 8
221521_s_at	8.813	8.111	7.219	7.902	7.804	7.820	8.802	8.152	0	-0.70	-1.59	-0.91	-1.01	-0.99	-0.01	-0.66	GINS2	GINS complex subunit 2 (Psf2 homolog)
221591_s_at	6.960	6.563	6.621	6.308	5.569	5.184	7.059	6.402	0	-0.40	-0.34	-0.65	-1.39	-1.78	0.10	-0.56	FAM64A	family with sequence similarity 64, member A
221727_at	8.174	7.489	7.196	6.892	6.422	6.122	7.495	7.080	0	-0.68	-0.98	-1.28	-1.75	-2.05	-0.68	-1.09	SUB1	SUB1 homolog (S. cerevisiae)
221965_at	6.639	6.588	5.147	5.638	5.184	4.571	5.368	4.906	0	-0.05	-1.49	-1.00	-1.45	-2.07	-1.27	-1.73	MPHOSPH9	M-phase phosphoprotein 9
222962_s_at	8.092	7.371	6.098	6.760	6.826	5.880	7.510	7.345	0	-0.72	-1.99	-1.33	-1.27	-2.21	-0.58	-0.75	MCM10	minichromosome maintenance complex component 10
223307_at	8.809	7.306	8.049	8.018	6.987	6.701	8.773	8.242	0	-1.50	-0.76	-0.79	-1.82	-2.11	-0.04	-0.57	CDCA3	cell division cycle associated 3
223570_at	7.905	7.210	5.835	6.478	6.248	4.916	6.897	6.729	0	-0.69	-2.07	-1.43	-1.66	-2.99	-1.01	-1.18	MCM10	minichromosome maintenance complex component 10
224428_s_at	8.911	8.644	7.664	7.841	7.308	7.784	8.918	8.890	0	-0.27	-1.25	-1.07	-1.60	-1.13	0.01	-0.02	CDCA7	cell division cycle associated 7
224509_s_at	7.013	6.703	6.014	6.083	5.930	4.618	4.322	4.207	0	-0.31	-1.00	-0.93	-1.08	-2.40	-2.69	-2.81	RTN4IP1	reticulon 4 interacting protein 1
224865_at	7.517	6.475	6.265	6.203	5.828	6.282	6.553	5.888	0	-1.04	-1.25	-1.31	-1.69	-1.24	-0.96	-1.63	FAR1	fatty acyl CoA reductase 1
225108_at	8.060	6.977	6.751	6.776	5.982	6.365	7.443	6.915	0	-1.08	-1.31	-1.28	-2.08	-1.70	-0.62	-1.15	AGPS	alkylglycerone phosphate synthase
225687_at	9.480	8.032	8.460	8.569	7.731	7.756	7.570	7.112	0	-1.45	-1.02	-0.91	-1.75	-1.72	-1.91	-2.37	FAM83D	family with sequence similarity 83, member D
226775_at	7.745	7.042	6.017	6.031	5.958	5.260	7.603	7.160	0	-0.70	-1.73	-1.71	-1.79	-2.49	-0.14	-0.58	ENY2	enhancer of yellow 2 homolog (Drosophila)
227164_at	6.554	6.797	5.812	4.702	4.904	5.250	5.462	5.250	0	0.24	-0.74	-1.85	-1.65	-1.30	-1.09	-1.30	SFRS1	splicing factor, arginine/serine-rich 1
227349_at	4.484	4.468	3.655	3.735	3.817	3.983	4.571	4.335	0	-0.02	-0.83	-0.75	-0.67	-0.50	0.09	-0.15	HELLS	helicase, lymphoid-specific
227350_at	8.317	7.563	6.931	7.040	6.526	7.467	8.032	7.588	0	-0.75	-1.39	-1.28	-1.79	-0.85	-0.28	-0.73	HELLS	helicase, lymphoid-specific
227578_at	5.117	2.987	3.224	4.150	3.131	2.841	3.459	3.184	0	-2.13	-1.89	-0.97	-1.99	-2.28	-1.66	-1.93	LOC100128191	hypothetical protein LOC100128191
227896_at	8.399	8.037	6.779	6.420	7.451	9.557	7.934	7.082	0	-0.36	-1.62	-1.98	-0.95	1.16	-0.46	-1.32	BCCIP	BRCA2 and CDKN1A interacting protein
228045_at	6.577	6.109	4.633	4.652	5.034	2.089	5.655	4.861	0	-0.47	-1.94	-1.93	-1.54	-4.49	-0.92	-1.72	---	---
228773_at	5.809	6.202	4.940	6.038	4.916	6.331	6.251	5.754	0	0.39	-0.87	0.23	-0.89	0.52	0.44	-0.05	---	---
228899_at	6.786	5.876	5.980	6.412	5.335	4.561	6.264	5.486	0	-0.91	-0.81	-0.37	-1.45	-2.23	-0.52	-1.30	LOC100132884	hypothetical protein LOC100132884
228987_at	7.273	7.791	5.020	5.658	5.622	6.251	7.484	6.659	0	0.52	-2.25	-1.62	-1.65	-1.02	0.21	-0.61	---	---
229126_at	8.323	7.835	7.692	8.282	7.540	4.993	7.183	6.339	0	-0.49	-0.63	-0.04	-0.78	-3.33	-1.14	-1.98	TMEM19	transmembrane protein 19
229129_at	7.559	6.015	6.702	6.930	5.835	7.457	7.493	7.037	0	-1.54	-0.86	-0.63	-1.72	-0.10	-0.07	-0.52	---	---
229305_at	6.502	7.096	5.057	5.191	5.241	6.203	6.928	6.530	0	0.59	-1.45	-1.31	-1.26	-0.30	0.43	0.03	MLF1IP	MLF1 interacting protein
232238_at	5.337	5.405	5.226	5.238	4.682	3.685	3.872	3.196	0	0.07	-0.11	-0.10	-0.66	-1.65	-1.47	-2.14	ASPM	asp (abnormal spindle) homolog, microcephaly associated (Drosophila)
235545_at	7.873	7.044	7.524	6.884	6.063	5.710	7.527	7.308	0	-0.83	-0.35	-0.99	-1.81	-2.16	-0.35	-0.57	DEPDC1	DEP domain containing 1
235572_at	7.880	6.249	6.614	6.997	6.916	6.036	6.287	5.317	0	-1.63	-1.27	-0.88	-0.96	-1.84	-1.59	-2.56	SPC24	SPC24, NDC80 kinetochore complex component, homolog (S. cerevisiae)
238431_at	7.430	7.173	6.452	6.410	5.886	4.789	6.257	5.710	0	-0.26	-0.98	-1.02	-1.54	-2.64	-1.17	-1.72	---	---
238529_at	4.346	3.082	3.511	3.575	3.550	2.627	3.440	2.538	0	-1.26	-0.83	-0.77	-0.80	-1.72	-0.91	-1.81	LOC730631	Hypothetical LOC730631
239002_at	6.266	5.934	6.982	7.146	5.541	4.737	6.246	6.096	0	-0.33	0.72	0.88	-0.73	-1.53	-0.02	-0.17	ASPM	asp (abnormal spindle) homolog, microcephaly associated (Drosophila)
239252_at	5.632	4.288	4.118	4.652	3.461	4.529	4.824	3.587	0	-1.34	-1.51	-0.98	-2.17	-1.10	-0.81	-2.04	COX7B	cytochrome c oxidase subunit VIIb
242300_at	6.230	5.989	4.625	5.036	4.255	3.018	5.255	4.038	0	-0.24	-1.61	-1.19	-1.98	-3.21	-0.98	-2.19	---	---
242890_at	6.949	6.202	5.260	5.440	4.938	6.355	6.759	6.290	0	-0.75	-1.69	-1.51	-2.01	-0.59	-0.19	-0.66	---	---
