# Supplementary material for: Compound C Prevents the Unfolded Protein Response during Glucose Deprivation through a Mechanism Independent of AMPK and BMP Signaling
Source: PLoS One. 2012 Sep 24;7(9):e45845. doi: 10.1371/journal.pone.0045845 (PMC3454318; doi:10.1371/journal.pone.0045845)
Supplement: Text S1 — Supplementary Methods. (PDF) [file pone.0045845.s008.pdf]

Supporting Information

Compound C prevents the unfolded protein response during glucose deprivation through a mechanism independent of AMPK and BMP signaling

Sakae Saito, Aki Furuno, Junko Sakurai, Hae-Ryong Park, Kazuo Shin-ya, and Akihiro Tomida

Materials and Methods

Immunoblot analysis

Cells were lysed in 1× sodium dodecyl sulfate (SDS) sample buffer (62.5 mM Tris-HCl, pH 6.8, 2% SDS, 5% 2-mercaptoethanol, and 10% glycerol), and protein concentrations of the lysates were measured with a Bio-Rad protein assay kit (Bio-Rad, Hercules, CA, USA). Equal amounts of proteins were resolved on a 4%–20% SDS-polyacrylamide gradient gel and transferred by electroblotting onto a nitrocellulose membrane. Immunoblots were probed with the following antibodies: mouse monoclonal anti-KDEL (to detect GRP78 and GRP94; StressGen, Victoria, BC, Canada), anti-4E-BP1, anti-phospho-4E-BP1 (Ser65), anti-LKB1, anti-AMPKα, anti-phospho-AMPKα (Thr172), anti-SMAD1, anti-phospho- SMAD 1 (Ser463/465) / SMAD 5 (Ser463/465) / SMAD 8 (Ser426/428) (Cell Signaling Technology, Inc., Danvers, MA, USA), rabbit polyclonal anti-ATF4 (Proteintech Group, Inc., Chicago, IL, USA), rabbit polyclonal anti-PERK (Abcam plc., Cambridge, UK), anti-ATF6 (BioAcademia, Osaka, Japan), anti-FLAG M2 (to detect FLAG-tagged ATF6 proteins; Sigma) and anti-β-actin (internal control; Sigma). The specific signals were detected with horseradish peroxidase–conjugated second antibody (Amersham Pharmacia Biotech, Tokyo, Japan) and a chemiluminescence detection system (PerkinElmer, Inc., Waltham, MA, USA).

RNAi experiments

Small interfering RNA knockdown experiments were performed with Stealth RNAi (Invitrogen). Stealth RNAi for SMAD1 are HSS106247, HSS106248, HSS180968. The Stealth RNAi negative control (Invitrogen) was used as an siRNA control. Transfections were performed using the Lipofectamine RNAiMAX transfection reagent (Invitrogen) with antibiotic-free RPMI-1640 medium supplemented with 5% FBS, according to the manufacturer’s protocol.

Cell viability assays

For the 3-(4,5-dimethylthiazol-2-yl)-2,5-diphenyltetrazolium bromide (MTT) assay, HeLa and 786-O cells were seeded in 96-well plates at 3×10^3 and 1.5×10^3 cells per well, respectively. After an overnight culture, cells are treated for 30 h with various concentrations of compound C, versipelostatin and phenformin in the presence or absence of 10 mM 2DG or 1 μg/mL of tunicamycin as a stressor. For the combination study, 786-O cells are treated for 24 h with various concentrations of compound C, versipelostatin, phenformin and buformin in the presence or absence of 10 mM 2DG. The medium was then replaced with fresh growth medium, and cells were cultured for a further 15 hours. Subsequently, MTT (Sigma) was added to the culture medium, and the absorbance of each well was determined as described previously [1]. Relative cell survival (mean ± SD of quadruplicate determinations) was calculated by setting each control absorbance from non–drug-treated cells as 100%. The effects of drug combinations at concentrations that produced 80% cell growth inhibition (IC_{80}) were analyzed using isobologram methods [2].

For the viability assay under glucose-withdrawal conditions, HT1080 cells were cultured overnight in 12-well plates (5×10^4 cells/well) and treated with various concentrations of compound C and phenformin in the presence or absence of glucose for 18 h. The cells were then treated with trypsin, seeded in 96-well plate with growth medium and cultured for a further 48 h. Subsequently, MTT was added to the culture medium, and the absorbance of each well was determined as described previously [1]. Relative cell survival (mean ± SD of quadruplicate determinations) was calculated by setting each control absorbance from non–drug-treated cells as 100%.

Reporter assay

Reporter assay was performed as described previously [3]. HT1080 and XBP1-Luc/HT1080 cells (3×10^5 cells/well) were cultured overnight in a 6-well plate, and transfection was performed. The cells were incubated for 8 h with a transfection mixture containing 500 ng of firefly luciferase–containing reporter plasmids (pGRP78pro160-Luc [3] or FLAG-tagged XBP1-Luc) and 1 ng of renilla luciferase–containing plasmid phRL-CMV (Promega, Fitchburg, WI, USA) as an internal control. XBP1-Luc/HT1080 cells were transfected with only phRL-CMV. The medium was then replaced with fresh growth medium, and the cells were incubated for another 4 h. Subsequently, the cells were reseeded in a 96-well plate (5×10^3 cells/well), cultured overnight, and treated for 18 h with various concentrations of compound C, versipelostatin, or phenformin with or without 10 mM 2DG or 5 μg/mL of tunicamycin. Relative activity of firefly luciferase to renilla luciferase was determined using the Dual-Glo Luciferase Assay System (Promega).

PCR analysis

HT1080 cells were seeded in 6-well plates (2 × 10^5 cells per well) and treated with compound C and versipelostatin in the presence or absence of 10 mM 2DG for 18 h. Total RNA was isolated from the cells using the RNeasy Mini Kit (Qiagen, Hilden, Germany) and converted to cDNA with SuperScript II reverse transcriptase (Invitrogen).

To detect XBP1 mRNA splicing valiant, we amplified each cDNA using a specific primer pair with Taq DNA polymerase (Roche) by incubation at 95ºC for 2 min, followed by 30 cycles each at 95ºC for 30 sec, 55ºC for 30 sec and 72ºC for 1 min. The primer pair used in the PCR was: Fw; 5′ - CCT TGT AGT TGA GAA CCA GG - 3′ and Rv; 5′ - GGG GCT TGG TAT ATA TGT GG - 3′ with amplicon sizes of 441 bp (unspliced form) and 415 bp (spliced form) [3]. Quantitative analysis of each PCR product was performed with a 2100 Bioanalyzer (Agilent Technologies, Santa Clara, CA, USA).

The quantitative real-time PCR was then performed with FAM-labeled D-LUX primer sets (Invitrogen), as described previously [4]. The specific primers of the XBP1 gene designed with the D-LUX Designer software (Invitogen) were: Fw; 5′ - CGC TGA GAA CCA GGA GTT AAG ACA GG - 3′, Rv; 5′ - GCC ACT GGC CTC ACT TCA TTC - 3′. Reactions were conducted in the ABI PRISM 7700 (Applied Biosystems, CA, USA). Fluorescence was monitored during every PCR cycle at the annealing step.

Microarray and statistical analysis

Details of the experimental conditions are provided as Table S1. Total RNA from HT1080 cells was isolated using the RNeasy RNA purification kit (Qiagen). The quality of total RNA was analyzed using the RNA 6000 Nano LabChip kit on a 2100 Bioanalyzer (Agilent). cRNA targets for hybridization to GeneChip were prepared by reverse transcription from 100 ng of total RNA. Targets were then labeled with biotin before fragmentation according to standard Affymetrix protocols. Hybridization to GeneChip Human Genome U133 Plus 2.0 arrays (Affymetrix) was carried out using the Fluidics Station 450 and GeneChip Scanner 3000 (Affymetrix). Normalization of microarray data was carried out using the Robust Multichip Average (RMA) method, RMAExpress version 1.0 [5]. Significantly expressed 8,778 probe sets were selected by the signal intensity (Log_2) > 6 in all 8 samples as a cut-off. The Glucose Deprivation Signature (UPR-related 246 probe sets) was previously identified [4]. Unsupervised, hierarchical clustering of signature genes was performed using Cluster 3.0 software and was visualized with Java TreeView [6].

References

1. Denizot F, Lang R (1986) Rapid colorimetric assay for cell growth and survival. Modifications to the tetrazolium dye procedure giving improved sensitivity and reliability. J Immunol Methods 89: 271-277.

2. Steel GG, Peckham MJ (1979) Exploitable mechanisms in combined radiotherapy-chemotherapy: the concept of additivity. International journal of radiation oncology, biology, physics 5: 85-91.

3. Park HR, Tomida A, Sato S, Tsukumo Y, Yun J, et al. (2004) Effect on tumor cells of blocking survival response to glucose deprivation. J Natl Cancer Inst 96: 1300-1310.

4. Saito S, Furuno A, Sakurai J, Sakamoto A, Park HR, et al. (2009) Chemical genomics identifies the unfolded protein response as a target for selective cancer cell killing during glucose deprivation. Cancer Res 69: 4225-4234.

5. Irizarry RA, Hobbs B, Collin F, Beazer-Barclay YD, Antonellis KJ, et al. (2003) Exploration, normalization, and summaries of high density oligonucleotide array probe level data. Biostatistics 4: 249-264.

6. de Hoon MJ, Imoto S, Nolan J, Miyano S (2004) Open source clustering software. Bioinformatics 20: 1453-1454.
